# Analysis of HIV-1 intersubtype recombination breakpoints suggests region with high pairing probability may be a more fundamental factor than sequence similarity affecting HIV-1 recombination

**DOI:** 10.1186/s12985-016-0616-1

**Published:** 2016-09-21

**Authors:** Lei Jia, Lin Li, Tao Gui, Siyang Liu, Hanping Li, Jingwan Han, Wei Guo, Yongjian Liu, Jingyun Li

**Affiliations:** Department of AIDS Research, State Key Laboratory of Pathogen and Biosecurity, Beijing Institute of Microbiology and Epidemiology, Beijing, 100071 China

**Keywords:** HIV-1, Genetic recombination, Reverse transcription, Branched structure, Mistemplating, the *intertwinement* model, DNA meiotic recombination

## Abstract

**Background:**

With increasing data on HIV-1, a more relevant molecular model describing mechanism details of HIV-1 genetic recombination usually requires upgrades. Currently an incomplete structural understanding of the copy choice mechanism along with several other issues in the field that lack elucidation led us to perform an analysis of the correlation between breakpoint distributions and (1) the probability of base pairing, and (2) intersubtype genetic similarity to further explore structural mechanisms.

**Methods:**

Near full length sequences of URFs from Asia, Europe, and Africa (one sequence/patient), and representative sequences of worldwide CRFs were retrieved from the Los Alamos HIV database. Their recombination patterns were analyzed by jpHMM in detail. Then the relationships between breakpoint distributions and (1) the probability of base pairing, and (2) intersubtype genetic similarities were investigated.

**Results:**

Pearson correlation test showed that all URF groups and the CRF group exhibit the same breakpoint distribution pattern. Additionally, the Wilcoxon two-sample test indicated a significant and inexplicable limitation of recombination in regions with high pairing probability. These regions have been found to be strongly conserved across distinct biological states (i.e., strong intersubtype similarity), and genetic similarity has been determined to be a very important factor promoting recombination. Thus, the results revealed an unexpected disagreement between intersubtype similarity and breakpoint distribution, which were further confirmed by genetic similarity analysis. Our analysis reveals a critical conflict between results from natural HIV-1 isolates and those from HIV-1-based assay vectors in which genetic similarity has been shown to be a very critical factor promoting recombination.

**Conclusions:**

These results indicate the region with high-pairing probabilities may be a more fundamental factor affecting HIV-1 recombination than sequence similarity in natural HIV-1 infections. Our findings will be relevant in furthering the understanding of HIV-1 recombination mechanisms.

**Electronic supplementary material:**

The online version of this article (doi:10.1186/s12985-016-0616-1) contains supplementary material, which is available to authorized users.

## Background

A prominent characteristic of the human immunodeficiency virus type 1 (HIV-1) is a high rate of recombination, resulting in increased genetic diversity and linkage of resistance mutations in the same genome. Recombination occurs frequently during reverse transcription, and can lead to a combination of beneficial mutations, the loss of deleterious mutations, or new starting points for subsequent viral evolution [1]. Currently, 72 HIV-1 circulating recombinant forms (CRFs), as well as a large number of unique recombinant forms (URFs) have been identified, both of which cause common infections in partial regions or around the world. Therefore, understanding the recombination process is important for unraveling the evolutionary history of HIV and explaining the pattern of HIV diversity [2].

HIV-1 is composed of two copies of positive single-stranded genomic RNA (gRNA). When a host cell is co-infected with two genetically distinct viruses, progeny viral particles with heterodimeric gRNAs would be produced. Upon subsequent infection of a new host cell by these mature heterodimeric progenies, recombination events during reverse transcription would result in a recombinant provirus [1]. Extensive experiments have been performed on HIV-1 as well as other retroviruses, all suggesting that HIV-1 genetic recombination results from a “copy choice” mechanism i.e. the alternating use of two templates during the synthesis of a single viral DNA strand [3]. With an ever increasing amount of data on HIV-1, the proposed genetic recombination mechanism of HIV-1 as well as a more representative molecular model requires repeated upgrades. For example, HIV-1 and other retrovirus recombination events have been predicted historically to follow the “*strand displacement-assimilation*” model [4, 5], the “*forced-copy-choice*” model [6], and, currently, the most favored “*minus-strand exchange*” model [7]. Thereunto, the specific events in the *minus-strand exchange* model involve initiation by nicking, acceptor invasion, primer strand realignment by branch migration, and template switching (Additional file 1: Figure S1). Donor-acceptor sequence similarity underlies primer strand realignment which is the most critical basis of the model.

Though HIV-1 recombination has been well established, there exist several serious concerns. E.g., (1) the lack of a complete structural understanding of how the RT structurally recognizes and recruits the acceptor template, to which RT transfers, and then releases the donor template, with which it is associated prior to template switching [1]. Additionally, (2), the manner in which reconciliation of conflicting data regarding the relationship between recombination and mutations would be achieved is still unclear. It is a common view that recombination breakpoints, within genomes, can indicate mechanistic details of the recombination process. Moreover, recombination hot and/or cold spots have been identified repeatedly either in experimental studies or in computational studies [8–12]. Given this, we set out to investigate the relationship between breakpoint distributions and (1) the probability of base pairing, and (2) intersubtype genetic similarities to further explore the structural details and molecular mechanisms of recombination events. A prominent feature of the present work is that it is based on near-full-length genome sequences of natural stains across large geographic regions (CRFs are from around the world, and URFs are from Asia, Europe, and Africa).

We greatly appreciate the contributions of those investigators whose extensive studies on HIV-1 recombination has provided the foundational data as well as the technical guidance upon which the discussion of our work is based.

## Methods

### Sequence collection

The following sequence sets were retrieved from the Los Alamos HIV sequence database (http://www.hiv.lanl.gov). Set 1: Near full length sequences (one sequence per patient) of URFs from 3 continents (according to *Definition of the geographic regions* in the HIV Databases) where there are prevalent recombinants circulating, including Africa, Asia, and Europe. Set 2: All available sequences of CRFs from around the world. For each CRF only the representative strain given in the database was included in the analysis. It should be pointed out there are not available sequences for CRF41 and CRF50 in the HIV Database. Additionally, the HIV Database does not provide representative references for CRF56, CRF58, and CRF60, thus we defined the following as references: accession numbers KC852173 for CRF56, KC522031 for CRF58, and KC899079 for CRF60, respectively. Table 1 details selected sequences information. All sequences used in this study are available on request.

**Table 1:** Information of selected sequences

Geographic regions	No. of collected sequences (Closing date)	No. of jpHMM-indicated recombinant sequences	No. of total breakpoints	Average breakpoints per genome
URFs-Africa	145 (March 20, 2014)	141	859	6.1
URFs-Asia	130 (March 16, 2014)	128	578	4.5
URFs-Europe	63 (March 17, 2014)	59	377	6.4
CRFs-global	59 (March 17, 2014)	57	323	5.7

### Recombination detection

The jumping profile hidden Markov model (jpHMM) [13] was used to identify the breakpoint locations in all collected sequences at a near full length level. As has been demonstrated in our previous study [14], jpHMM can easily obtain highly accurate recombination data. The recombination prediction in jpHMM is based on a precalculated multiple sequence alignment of the major HIV-1 subtypes including CRF01_AE references, and the evaluation of its prediction accuracy showed that it is more accurate than the competing methods used for phylogenetic breakpoint detection [13, 15]. In jpHMM, each HIV-1 subtype is represented by a profile hidden Markov model and all profile models are connected by empirical probabilities. For the identified recombinants, the tool can provide detailed subtype composition and well resolved breakpoint locations [13, 15]. After recombination detection, recombination breakpoints presented in incremented windows of 100 nucleotides were numbered. Subsequently, 1/3 of the average value of a total breakpoint frequency along 86 windows was defined as the cold spot boundary line and 5/3 of the average value was defined as the hot spot boundary line. Details of all recombination patterns in this study are available on request.

### RNA secondary structure data

RNA secondary structure data was based on the NL4-3 HIV-1 genome (Genbank accession number AF324493) structure [16]. In this work, a pairing probability was assigned at single nucleotide resolution *via* examining evolutionary information contained in nucleotide and amino acid variation to infer RNA structure [16]. The scores quantifying the degree of base pairing at each site along the HIV-1 genome were downloaded from the journal website (http://www.nature.com/nature/journal/v460/n7256/suppinfo/nature08237.html). Although these data are from the NL4-3 strain, it was the best and the only analytical choice at the present time and have been cited widely as the representative structure of the HIV-1 M group or CRFs [11, 17].

### Similarity analysis

Intersubtype genetic similarity was calculated as the mean pairwise-intersubtype sequence identity in the 100 nucleotide windows across an alignment. The alignment is composed of 9 subtype representative sequences of HIV-1 group M. All nine sequences are from the recombination identification program (RIP) alignment in the HIV Database (http://www.hiv.lanl.gov/content/sequence/NEWALIGN/align.html#RIP). A-J are the consensus sequences and K is a representative sequence (accession number AJ249235).

### Statistical analysis

A Pearson correlation test was used to compare correlations of breakpoint distribution between different-continent-derived URF and CRF groups. In addition, a Pearson correlation test was also performed to test the comparison of genetic similarity to the breakpoint distribution. The Wilcoxon two-sample test was performed to compare pairing probability within cold spot regions to those within hot spot regions. SAS, version 9.2 (SAS Institute), was used for all analyses.

## Results

### URF groups from Asia, Africa, Europe and CRF group from around the world

**Fig. 1:**
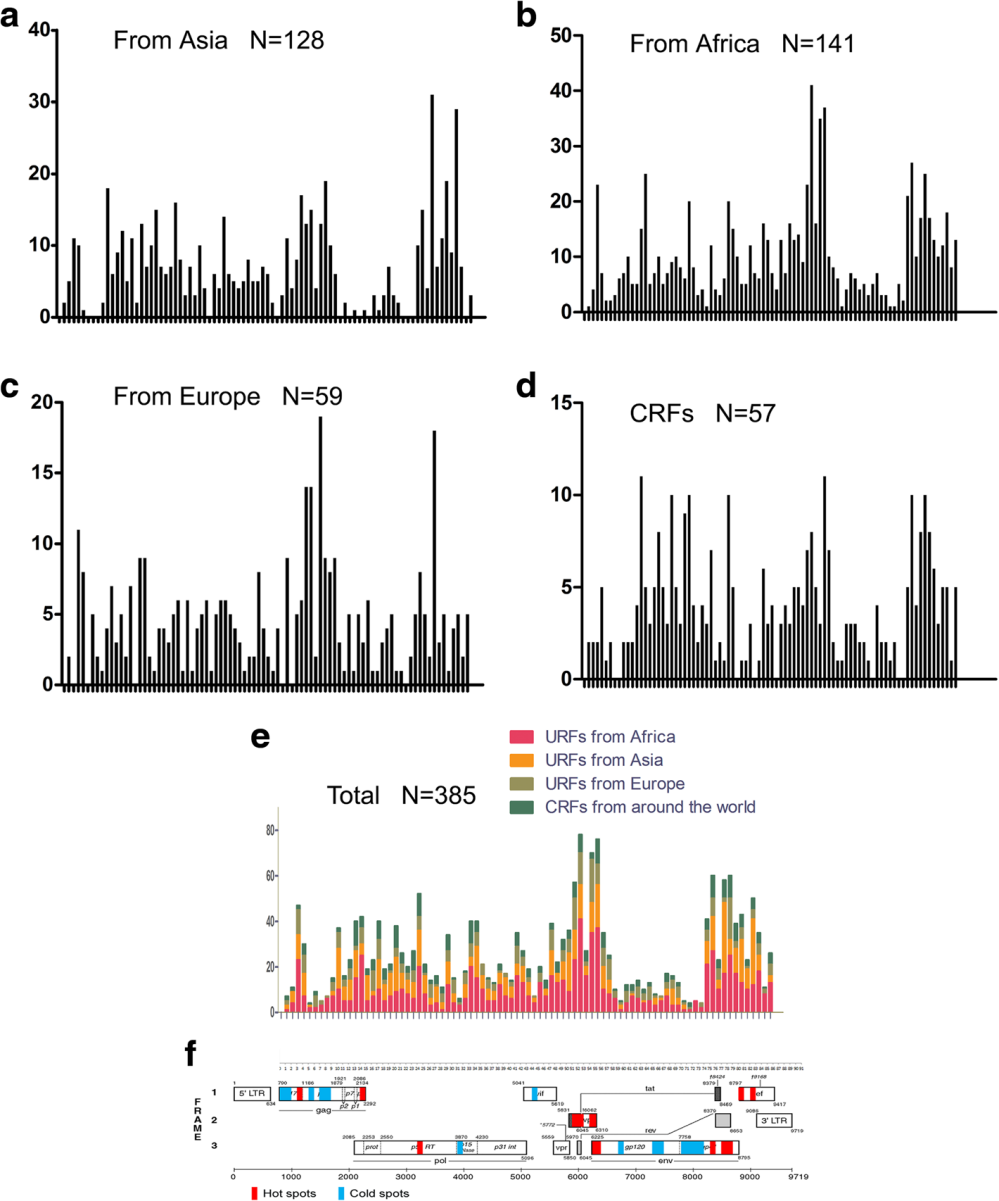
Breakpoint frequency in near full-length URF and CRF groups. **a** The breakpoint distribution of URFs from Asia. **b** The breakpoint distribution of URFs from Africa. **c** The breakpoint distribution of URFs from Europe. **d** The breakpoint distribution of global CRFs. **e** The integrated distribution of the 4 groups. **f** The hot regions and cold regions and corresponding gene locations. The breakpoint positions are based on the HXB2 numbering (from HXB2 790 nt to 9417 nt). Horizontal blue lines: average frequency value per window; horizontal red lines: hot spot boundary line; horizontal black lines: cold spot boundary line. Frequency scales are some different in each panel for better resolution

The sequence information of CRFs, from around the world, and that of URFs from Asia, Africa, and Europe, are provided in Table 1. The breakpoint distributions for the CRF and URF groups were calculated using non-overlapping 100 nucleotide windows (86 windows in total from HXB2 790 nt to 9417 nt) respectively (Additional file 1: Table S1). A Pearson correlation test was performed to test whether there were differences in the breakpoint distributions between different groups. The results indicated all the URF and CRF groups exhibited the same distribution with similar peaks and valleys (Fig. 1a-d, Table 2). The recombination breakpoint distribution across the whole genome was identical to previously published analysis [8], except that a 100-nt instead of 200-nt/500-nt window was used to calculate breakpoint clustering. The results indicated that a recombination pattern was not a key factor impacting a recombinant virus becoming CRF or URF. In addition, considering significant diversity of sequences in HIV-1 subtypes, human species, and geography, such breakpoint distribution patterns were likely due to mechanistic processes that transcend HIV-1 subtypes, human species, and geography.

**Table 2:** Pearson correlation test results of breakpoints distribution of all 4 groups

	URFs from Asia	URFs from Europe	CRFs-global
URFs from Africa	*r*: 0.54533 p: <.0001	*r*: 0.68364 *p*: <.0001	*r*: 0.64156 *p*: <.0001
URFs from Asia		*r*: 0.34818 *p*: 0.0010	*r*: 0.58301 *p*: <.0001
URFs from Europe			*r*: 0.42501 *p*: <.0001

These four groups (one CRF group and three URF groups) of recombinant data were then integrated into one (Fig. 1e). The Run test indicated recombination breakpoints are not distributed randomly across the HIV-1 genome (the Run (median) test, Z: -3.905, *P*: .000; the Run (me﻿an) test, Z: -2.750, *P*: .006﻿﻿. A Chi-squared goodness of fit test can also be performed to test the random of the distribution as described previously [8]). Thus the analysis provided significant evidence of recombination “hot spots” as well as regions where recombination was limited across the HIV-1genome (cold spots). The average value for a total breakpoint frequency was 24.85 per window. In this study, 1/3 of the average value, 8.28, was defined as the cold spot boundary line and 5/3 of the average value, 41.41, was defined as the hot spot boundary line (i.e., 24.85 ± 16.57). The hot regions and cold regions, as well as corresponding gene locations, are summarized in Additional file 1: Table S2 and Fig. 1f.

### Evaluation of the trend of recombination in regions with high pairing probability

The incorporated data yielded a total breakpoint distribution (Fig. 1e). According to the definition of cold spot and hot spot boundary lines, we found hot spot regions harbored in 1200 nucleotide sites and cold spot regions harbored in 1400 nucleotide sites (Additional file 1: Table S2). In one key published study, pairing probability was assigned for each nucleotide based on a single-nucleotide resolution analysis of a complete HIV-1 NL4-3 genome [16]. This provided a rare opportunity to test the influence of an RNA stem structure on HIV recombination breakpoint distributions. The Wilcoxon two-sample test indicate the pairing probability within cold spot regions is significantly higher than within hot spot regions (Z = −5.1330, Two-Sided *p* < .0001, Additional file 1: dataset 1 and 2). This result indicated a significant trend; more recombination events occurring within single-stranded regions, and limited recombination events occurring in double-stranded regions.

This preference is rather inexplicable, given the discovery that regions with high pairing probabilities within a single strand (likely to be double-stranded stem regions), were strongly conserved across distinct biological states [16, 18]. It is well known that strong conservation implies good intersubtype similarity. Additionally, genetic similarity has been shown to be a very critical factor promoting recombination [19]. Therefore, in light of the study by An et al. [19], we expected that our results should have shown that more recombination events would be found to occur in double-stranded regions. Thus, the current analysis revealed an unexpected disagreement between intersubtype similarity and the breakpoint distribution.

### Intersubtype genetic similarity

In order to further validate the results above, we performed a comparison of intersubtype genetic similarity and the breakpoint distribution, in 100 nucleotide non-overlapping windows, to directly test their correlations. The mean pairwise-intersubtype sequence identity in the 100 nucleotide windows between 9 subtype representative sequences for the HIV-1 group M was defined as genetic similarity (Additional file 1: Table S3). The test result was not significant (Pearson correlation, *r* = −0.08062, d.f. = 86; *P* =0.4606 > 0.05). These results further indicated as the analysis aforementioned did, an unexpected disagreement between the breakpoint distribution and genetic similarity.

These contradictory results indicate a conflict that cannot be ignored. Donor-acceptor similarity has been compellingly reported to be an important factor promoting recombination [19]. Moreover, it underlies nascent DNA strand realignment which is the critical basis of the currently favored “*minus-strand exchange*” model [7] (Additional file 1: Figure S1). The current results, however, did not show, as they were expected, that better similarity led to enhanced recombination. This surprising outcome suggested that more fundamental factors than sequence similarity, affecting recombination in natural conditions, exist.

## Discussion

The important study by An et al. provides key support for the role of sequence similarity inducing HIV-1 recombination. In their experimental system, the effects of systematically varying percentages of sequence similarity on HIV-1 recombination were examined using a repeat deletion assay [19]. This system was based on observations that repeated sequences were often deleted from retroviral vectors [20]. The results indicated a 5 % difference decreased the deletion frequency to 65 % of that for identical repeats, with recombination declining further as more variation was introduced. When repeats differed by 27 %, recombination was below the detection threshold, suggesting template switching was reduced more than 300-fold [19]. These compelling results indicated the essential role of intersubtype genetic similarity in promoting recombination.

In light of our unexpected findings, multiple discussions were conducted to confirm these results among the authors. And a consensus was reached that the apparent conflict between our current findings and previous studies were not essentially contradictory, but were rather strong indications that reflected the different experimental contexts. The experimental system used by An et al. employed HIV-1-based assay vectors rather than natural isolates, and thus there was only one experimental factor that is the varying percentages of sequence similarity. Conversely, our analysis was based on natural HIV-1 strains. Two previous studies that focused on HIV-1 gRNA [16, 18], reported that in nature, regions with high pairing probabilities (stem regions) closely corresponded to regions with good intersubtype similarity (strongly conserved regions). Therefore, in our analysis, although the purpose was also to test the association between sequence similarity and recombination frequency, there were actually two experimental factors, including both sequence similarity and stem structures due to their close binding. The apparent contradiction between recombination breakpoint distributions and genetic similarity presented in the current work exactly suggested that the stem regions can affect the occurrence of recombination. Thus, the present work does not overtly refute previous results regarding similarity inducing recombination. Instead, our study makes progress in fully understanding recombination in HIV-1. The current results indicate the stem structure (playing an inhibition role) to be a more fundamental factor than sequence similarity (playing a promotion role) affecting HIV recombination in nature.

In summary, the use of published data is an ideal method to address gaps in scientific research, and the availability of HIV sequence database (http://www.hiv.lanl.gov) is a great place to start. To further the understanding of HIV-1 recombination, we carried out a bioinformatics analysis of previously published URFs and CRFs. Through analysis, we confirmed an apparent conflict between the recombination breakpoint distribution and genetic similarity. This present work is based on near full length genomes of natural isolates. Therefore, contrary to results from experiments simulated in vitro, these results expose a deeper biological complexity than previously reported.

Notably, a previous study reported results that indicated a similar conflict. Simon-Loriere et al. reanalyzed independent data defining (1) the structure of a complete HIV-1 RNA genome and (2) favorable sites for recombination in the entire *env* gene and found sequence similarity was clearly not the only determinant for recombination breakpoint distribution, as recombination rates varied widely even within gene regions with high degrees of sequence identity [11]. This indication, however, did not draw substantive attention.

Finally, we conclude that the stem structure may be a more fundamental factor affecting HIV-1 recombination than sequence similarity underlying primer strand realignment which is the most critical basis of the “minus-strand exchange” model. The current analysis indicated that the conflict between results from natural HIV-1 isolates and those from HIV-1-based assay vectors does not overtly refute previous results regarding similarity inducing recombination, but indicates a deeper understanding of previous results through an analysis with more realistic situation. In addition to this unexpected conflict between intersubtype similarity and the breakpoint distribution, the analysis performed in the current work suggested a second concern: less recombination events occurred within double-stranded regions than within single-stranded regions, and what is the reason for this surprising preference? Neither of these concerns can be reasonably addressed within the “*minus-strand exchange*” recombination model; thus necessitating further exploration of the structural details and molecular mechanisms that govern HIV-1 genetic recombination. Therefore, we attempt to make somewhat revisions to this model and propose a speculative one for discussion.

### Model revisions: a branched structure could be one structural basis for HIV-1 recombination via mediating mistemplating

The revised model is a designated *intertwinement* model. The salient features of this model are: (1) the existence of a specific cross-linked branched structure (BS) of two nucleic acid strands prior to template switching, (2) the supplementation of template switching occurring at a joint position in addition to the previous view that recombination is from a discrete position, (3) the BS causes mistemplating of the RT thereby leading to recombination.

**Fig. 2:**
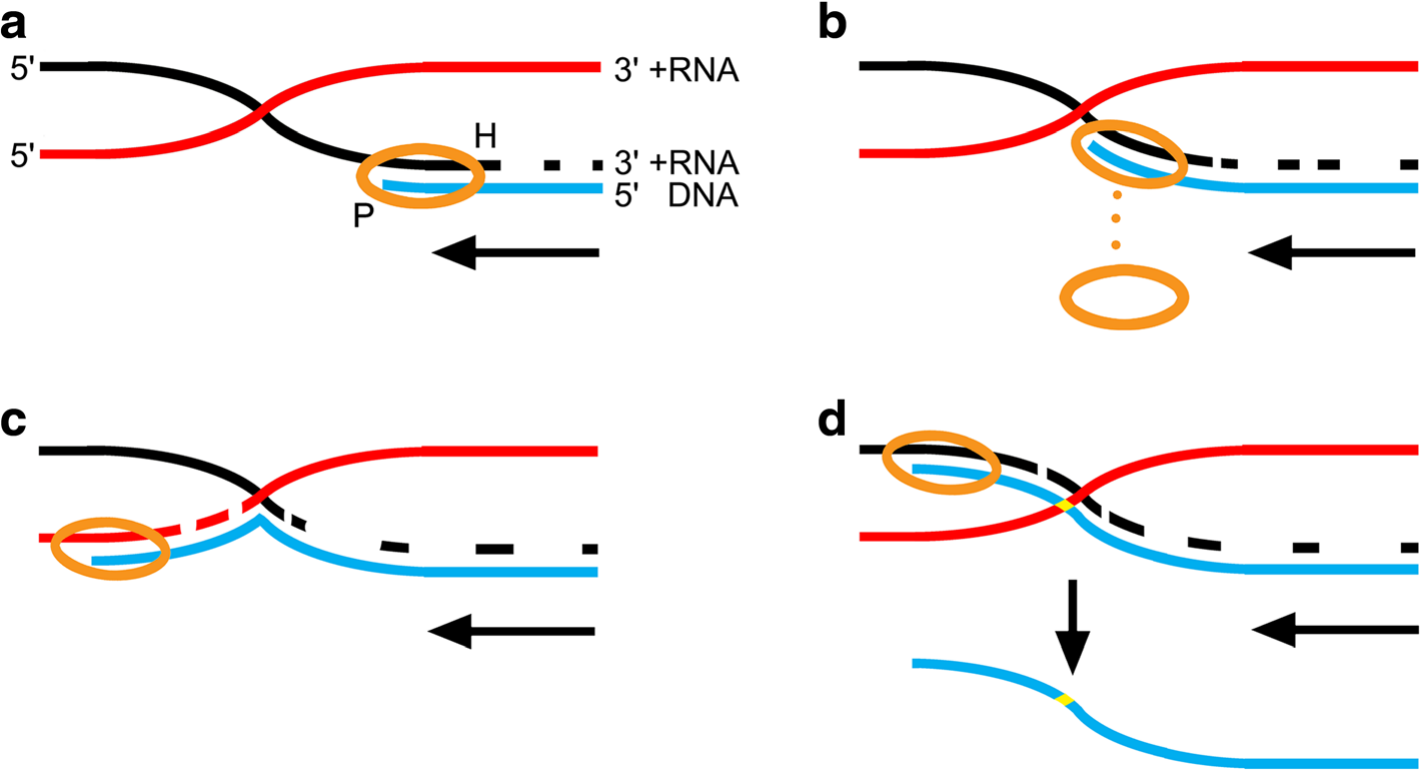
A BS and BS-based different results. **a** Relative motion between gRNA strands favors topological intertwining, leading to a junction containing both donor and acceptor templates, i.e., a BS. **b** During reverse transcription, RT pauses as a result of an impediment from the BS. Unable to proceed, reverse transcriptase is halted. **c** When RT encounters a BS, it “climbs up” the acceptor template and resumes the subsequent synthesis along the acceptor template, resulting in successful strand transfer. **d** When RT encounters a BS, it “climbs up” the acceptor template, performs a very brief synthesis of several bases along the new template, then “climbs down” back to the donor template, and continues elongating along the initial donor template. This will lead to recombination of a very small segment. Such a segment of gene variations would be regarded as serial mutations rather than recombination because it is too short to be detected by any of the recombination analysis programs including Simplot, Recombination identification program (RIP), jumping profile hidden Markov model (jpHMM), and RDP3. As shown in the panel, the allele on the red strand is preserved in the integrated provirus, resulting in mutations (the mutations are represented by the yellow site). HIV-1 RT is represented by the gold oval, where P indicates the DNA polymerase active site and H indicates the RNase H active site. The DNA polymerase active site is engaged at the primer strand 3′ terminus, and the RNase H active site engages the template strand and performs limited template degradation as DNA synthesis proceeds. The black lines represent donor templates; the red lines represent acceptor templates. The blue line represents a nascent DNA. The level arrow indicates the direction of DNA synthesis

HIV-1 recombination is a biochemical process that occurs in an intracellular and physical environment. Thus, understanding some fundamental physical properties, such as the relative motion between RNA strands, is critical to understanding the actual mechanism of recombination. A BS can possibly form during certain phases in the virus replication cycle; before reverse transcription and after synthesis of gRNAs from the provirus. This is due to the random relative motion between the gRNA strands that can result in topological intertwinement of strands and thus BS formation. A BS is a joint complex (junction) composed of two cross-linked templates: a donor template and an acceptor template (see Fig. 2a). It is like a joint molecule.

**Fig. 3:**
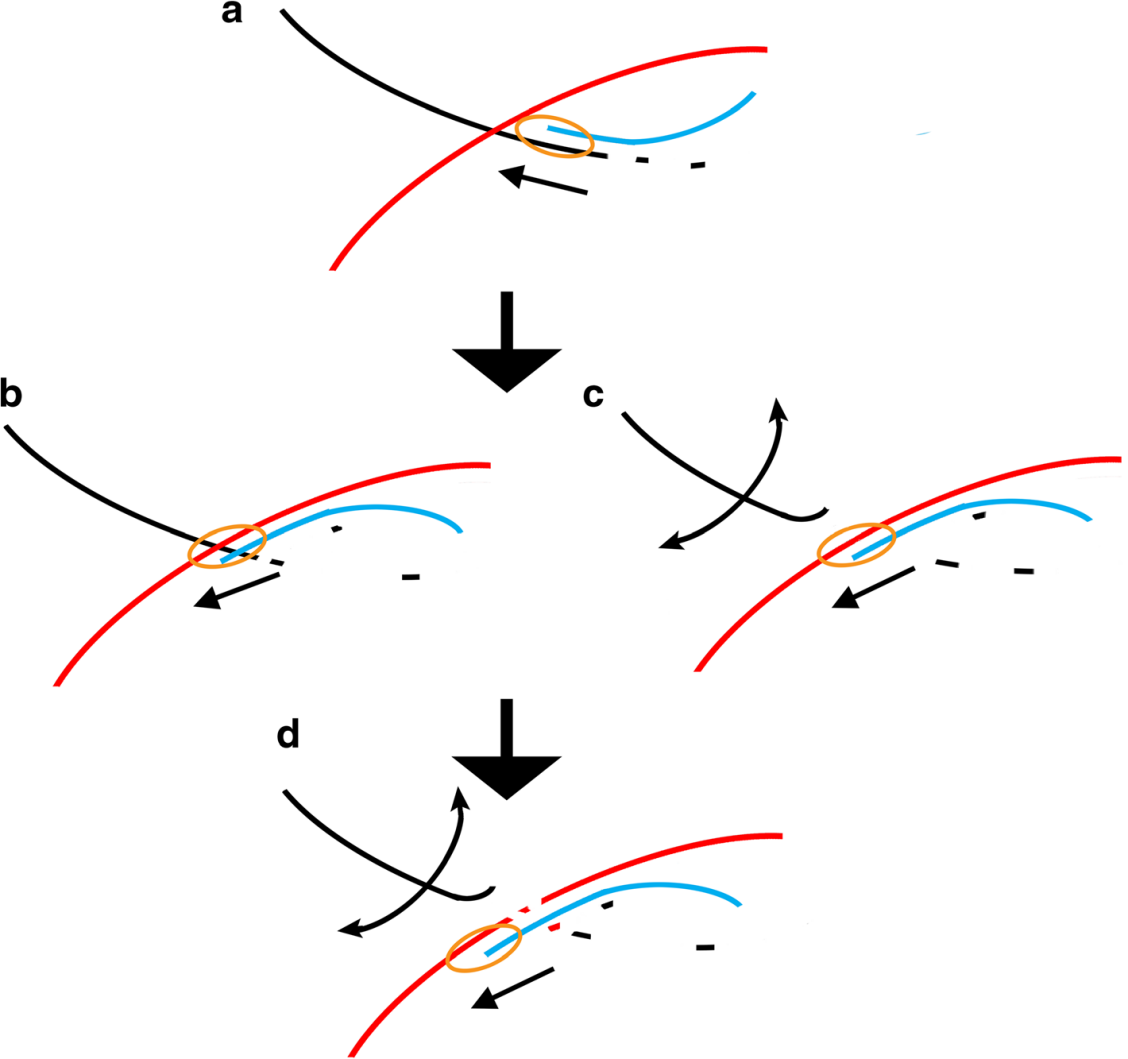
A detailed process of BS-based template switching interpreting HIV-1 homologous recombination. **a** RT encounters a BS during reverse transcription. Then, a “climb process” occurs. **b** When RT “climbs up” and transfers to the acceptor template, the realignment of the nascent DNA strand onto the acceptor template via base pairing behind the growing site would modulate forward direction of RT as a tadpole changes its direction by modulating the azimuth of its tail. This action would prevent RT from proceeding on in the initial direction, and adjust RT to advance along acceptor template. **c** Concomitantly, the already formed BS would be released more easily. The sway of two gRNA strands as well as the nascent DNA is inevitable under the kinetic actions. Therefore, losing the balance due to RNase H-mediated degradation, the donor template would readily dissociate with the acceptor template and the nascent DNA, resulting in the resolution of the BS. Unable to return to the donor template, RT “has to” continue the following synthesis along the acceptor template. **d** Finally, a successful homologous template switching is completed. The thin linear arrows indicate the direction of DNA synthesis. The arc arrows indicate the sway of the donor template

According to the *intertwinement* model, the RT will undergo a climb-like process when encountering a BS. This “climb process” is analogous to a snail “climbing” when encountering a rope that is blocking its path. Various outcomes can be generated under different situations (Fig. 2b-d). If a BS has a proper spatial configuration and is sufficiently stable, it can lead to mistemplating of RT during reverse transcription, and a recombination event arises (Fig. 2c). When three well investigated recombination factors are considered, i.e., extent of donor-acceptor sequence similarity [19], donor-acceptor homology length [21–23], and base pairing in complementary regions between the nascent DNA strand and the acceptor template at a region downstream of the crossover site [7], the *intertwinement* model shows even greater promise in interpreting homologous recombination (Fig. 3). It is important to point out that the RT does binds to a duplex, which is then composed of DNA and an acceptor, when transferring to the new template at a BS, rather than accommodating a triple-stranded structure composed of donor template, acceptor template, and nascent DNA. The *intertwinement* model can provide a clearer structural basis for the copy choice mechanism than previous molecular models.

### Difficulty in forming branched structures within double-stranded regions can lead limitation of recombination in these regions

According to the *intertwinement* model, a BS is composed of 2 single strands, and can be generated more readily between two single strands (inter-strands) or within a single strand (intra-strand). That is to say, double-stranded regions (like stem structure) along genomes have more difficulty in forming a BS. Understandably, fewer BS mediated recombination events occur. This scenario provides an explanation for the second concern raised: the underlying reason for recombination limitation in regions with high pairing probability.

Compared to the previous reports, a stem structure is a more fundamental factor than sequence similarity to genetic recombination in the *intertwinement* model because it can affect BS formation. Donor-acceptor similarity at regions where there can first form a BS would actually play roles in promoting recombination. This scenario can provide an interpretation for the first concern raised: the unexpected conflict between intersubtype similarity and the breakpoint distributions in actual incidences of the natural virus.

### The *intertwinement* model can provide new interpretations for the conflicting relationship between recombination and mutations

Whether genetic recombination and mutations occur independently or are mechanistically linked is a hot research topic. One viewpoint is that recombination can inhibit mutations during synthesis. Experiments performed in both MLV and HIV-1 showed that when RNase H activity was reduced, recombination was decreased, whereas DNA synthesis infidelity was drastically enhanced [24, 25]. Other studies reported results that support an opposing view. A recently published study reported a significantly higher mutation rate around the recombination site, and these occurrences were not simply co-localized in the genome [26]. It has also been reported that upon template switching, RT is quite capable of significant mismatch extension in both *in vitro* and viral replication [22, 27, 28]. Moreover, whenever clustered substitutions were observed, variations arose, most likely, via recombination rather than by serial point mutations [29, 30]. Taken together, these results indicate that genetic recombination might contribute to HIV-1 mutations.

What is the actual mechanistic relationship between recombination and mutations? As shown in Fig. 2c-d, the revised *intertwinement* model not only presents a mechanism for recombination, but also offers a mechanism by which mutations may occur. In this model one scenario can result in recombination (Fig. 2c) and an alternative scenario can result in “mutations” (Fig. 2d). Both outcomes can occur via the same mistemplating mechanism. The difference lies in length of replication after mistemplating. The recombination event occurs from a long segment of replication (Fig. 2c). While the scenario in Fig. 2d, refers to recombination events of very short replication, along the acceptor template (several bases). Such gene variation can be regarded as serial mutations rather than recombination, because the segment of variation is too short to be detected by any of recombination analysis programs at present [14]. Specifically, RNase H activity is a key factor involved in these two different results. RNase H mediated donor template degradation provides opportunities for base pairing between the acceptor template and the nascent DNA [7]. Furthermore, the *intertwinement* model indicates donor template instability, from RNase H mediated degradation, can promote the release of a BS (for details see Fig. 3c). Therefore, if the activity of RNase H is inhibited, the donor-DNA hybrid remains stable and preserved. First, this means that even if a homologous sequence exists, the nascent DNA has no chance of realigning with the acceptor template via base pairing. Additionally, it means the BS would remain stable (Additional file 1: Figure S2). Thus, the two key routes illustrated in Fig. 3b-c that govern recombination scenarios would not function. As a result, recombination would be restricted while mutation events are promoted. When RNase H activity is normal, recombination events would be facilitated and increased, while mutation events would be restricted. Thus, there appears to be an inversely proportional relationship between recombination and mutations.

Experimental data from King et al. provides further support for recombination inhibiting mutations. In this study, HIV-1 virions were engineered to contain only one single intact gRNA to prevent recombination, in the absence of a second template. These results showed that haploid reverse transcription was at least three-fold more error prone, and marker genes were inactivated by either point mutations or deletions [31]. This data could also be interpreted by the intertwinement model as follows. A BS does not only form between two strands, but can also form within a single strand (Intrastrand BS is shown in Additional file 1: Figure S3A). The same RT climb-like process can also occur at an intrastrand BS as at an interstrand BS, and thus result in gene deletion (Additional file 1: Figure S3B). By contrast, interstrand intertwinement can play a button-like role (Additional file 1: Figure S3C), stabilizing the two strands and decreasing intrastrand BS formation. The measures that HIV-1 virions were engineered to contain only one single intact gRNA to prevent recombination did not prevent all recombination, but just interstrand recombination. The same mechanism for recombination can also occur within one gRNA via intrastrand mistemplating. For example, as shown in Additional file 1: Figure S3A and B, the model depicts a portion of a viral gene being deleted via presenting an intrastrand BS mediated copy choice.

The opposing viewpoint is that genetic recombination contributes to HIV-1 mutations because more mutations are observed around the site of recombination. Within the revised model, multiple intertwinements can lead to clustered BSs (Additional file 1: Figure S4A). In this scenario, a serial climb process would be involved, during the reverse transcription, along such a region. If strand transfer happened successfully after multiple climbs (i.e., RT finally elongating along red strand), a crossover site as well as a surrounding cluster of mutations would be observed (Additional file 1: Figure S4B). Alternately, if strand transfer does not occur (i.e., RT finally still elongating along black strand), only clustered mutations would be observed (Additional file 1: Figure S4C).

## Conclusions

This study performed an analysis of the relationship between breakpoint distribution and (1) probability of base pairing, and (2) intersubtype genetic similarity. One possible limitation of the current work is the non-complete exclusion of selective pressure occurring within infected populations that may favor the outgrowth of certain recombination events over others. Although the significant diversity of sequences in HIV-1 subtypes, human species, and geography have suggested the observed uniform breakpoint distribution patterns are very likely due to mechanistic processes relative to selection, a re-performance based on isolates all derived from cell culture in vitro may be necessary, which would be an interesting research topic to explore.

Subsequently, a revision to the previous recombination model is also discussed here. The results and discussion indicated that both analysis and revisions are of great necessity. Specifically, more direct evidence for the *intertwinement* model also exists. Peliska et al. once detected a pre-switch complex that includes both donor and acceptor templates [32]. These results provide some support for the prediction of a BS as well as its existence prior to template switching of RT. In addition, experimental data from Abbondanzieri et al. suggested that elongating HIV-1 RT is much more of a molecular contortionist than previously recognized [33]. RT has high flexibility and can rapidly flip between alternate binding orientations for plus-strand primer generation and primer utilization. This result greatly extended our knowledge of previously established properties of elongating RT. Moreover, nucleocapsid (NC) can function as a molecular lubricant for elongating RT, because NC has been shown to promote RT elongation and reduce self-priming and pausing at template structures [34, 35]. Both features provide strong support for RT’s capacity to maneuver past a BS. The results and subsequent discussion of the model from the present work will be critical to further understanding of a more detailed recombination mechanism, as well as its role in the complex and dynamic evolution of HIV-1.

In summary, this revised mechanism depicts a more natural process based on objective factors. More biochemical and biophysical experiments are necessary to further determine the actual mechanism. The most required experiment could be 1) observation of the existence of a branched structure ahead of RT, 2) direct detection of the proposed RT climb process when it encounters a BS, and 3) identifications of factors that contribute to a BS, e.g., the HIV sequences surrounding the BS. Additionally, the intertwinement model not only presents a mechanism for recombination, but also offers a mechanism by which mutations may occur. Both outcomes can occur via the same mistemplating mechanism. The difference lies in length of replication after mistemplating (Fig. 2c-d). If this were true, the nature of this kind of mutations should match the genetic polymorphisms found on the acceptor RNA. Eventually, a detailed process could be reconstructed.

**Fig. 4:**
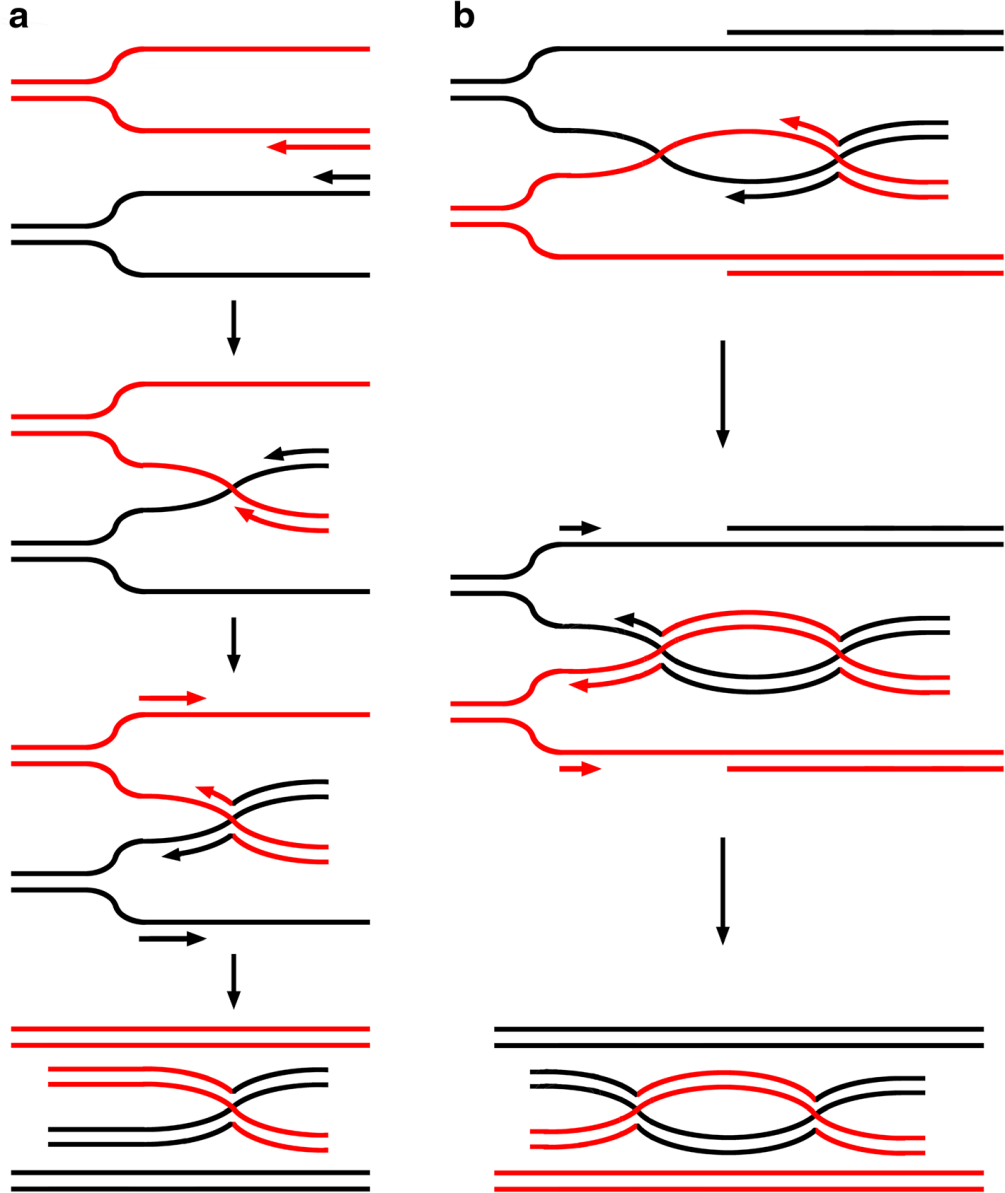
Holliday junctions (HJ) that arise from BS-based copy choice. **a** During DNA synthesis, a BS forms between two single-stranded DNAs of the same polarity. When the BS is encountered consecutively by the two polymerases, which engage maternal and paternal DNA, respectively, it mediates two rounds of copy choice, resulting in a single HJ. **b** Another BS forms downstream the first HJ. When both maternal and paternal polymerases encounter a second BS, the same process of copy choice is performed as at the first BS, leading to a double-Holliday junction

We acknowledge that the *intertwinement* model may also have implications regarding the basic mechanism underlying DNA meiotic recombination. The reasons are as follows; first, there are key similarities between HIV-1 reverse transcription and DNA replication (i.e., both require nucleic acid strand synthesis based on another nucleic acid template). Secondly, initial HIV-1 recombination model benefited from DNA meiotic recombination model. As the Fig. 4a shows, 2 rounds of such copy choice at the same BS would result in a four-stranded structure, which is exactly a Holliday junction (HJ) [36]. In contrast to the DSBR model where a double-Holliday junction (dHJ) is expected to arise from a double strand break (DSB), capture of the end, DNA synthesis, and ligation, a dHJ can form via a double-BS as detailedly shown in Fig. 4b.

**Fig. 5:**
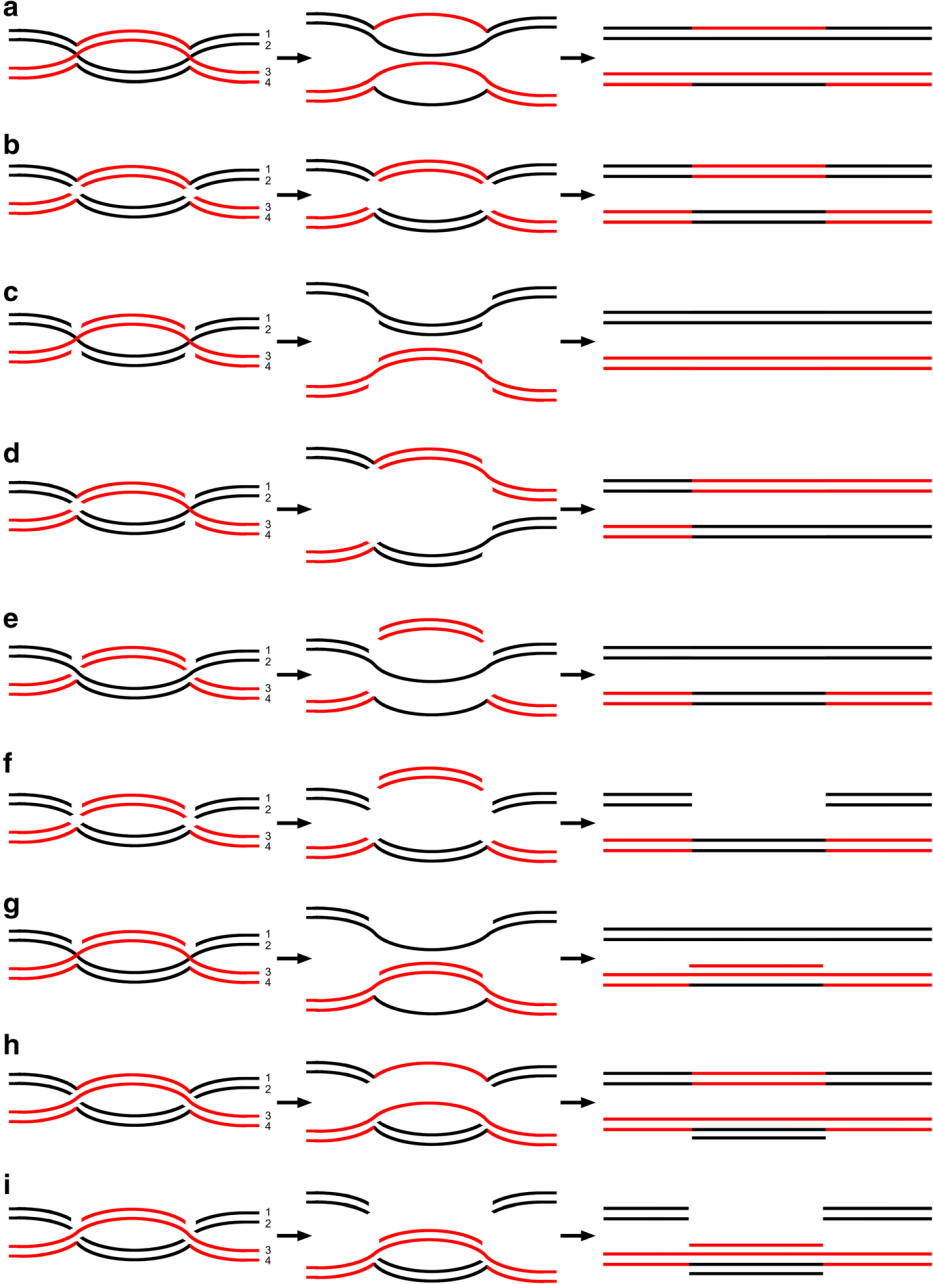
Various resolution patterns can lead to different DNA meiotic recombination events. **a** Meiosis I spindles begin to pull maternal and paternal chromosomes in opposite directions before both the HJs are resected. Thus, topologically, the dHJ is resolved and 2 symmetric heteroduplexes are generated. After subsequent mitosis, post-meiotic segregation occurs, displaying aberrant 4:4 segregation. **b** Resolution of 2 junctions by cutting inner, crossed strands leads to crossover of genes between 2 HJs. **c** Resolution of 2 junctions by cutting outer, noncrossed strands does not impact the products after repair synthesis. **d** Compared to (**b**), opposite-sense cutting, e.g., in the left-hand HJ, the crossed strands are cut, and in the right-hand HJ, the noncrossed strands are cut, generates crossover containing upstream regions. **e** Cuts are made on strand 1 and strand three in both HJs. After the pull of meiosis I spindle, 2 duplexes form, each of which contains a single-strand gap. Both gaps are subsequently repaired by repair synthesis, leading to gene conversion (6:2 segregation). **f** Cuts are made on strand 1, 2, and 3 in both HJs. After the pull and repair synthesis, the information on black chromatid is transferred to the homologous region of the red one. The original information on the black chromatid is deleted, leading to gene conversion of deletion. **g** Cuts are made on strand 1 in both HJs. The resolution generates a duplex with a single-strand gap and another duplex with a 3-stranded region. Within the 3-stranded region, there first is a duplex composed of strand 1 and strand 3. Then, the duplex interacts with strand 4, forming a 3-stranded DNA. After mitosis, the 3-stranded helical DNA results in post-meiotic segregation, displaying normal 5:3 segregation. **h** Contrary to (**g**), cuts are made on strand 2 in both HJs. Post-meiotic segregation also arises, but the products display aberrant 5:3 segregation. **i** When cuts are made on strand 1 and 2 in both HJs, the information on black chromatid would be deleted and transferred to the red one. Then, a 4-strand helical DNA composed of 2 duplexes appears. After mitosis, the 4-strand helical DNA can also result in post-meiotic segregation

A HJ is composed of 4 component single strands, according to upgraded understanding, if a HJ is resected stochastically by endonuclease digestion, the resolution should be variable. For example, any 1, any 2, any 3, all 4, or none of them can be digested. Therefore, there should be a total of 16 possible resolutions of each HJ, and thus 256 possible resolutions of each dHJ in theory rather than the only 4 patterns proposed previously [37]. Nine possible patterns are listed explaining observations including crossing-over, gene conversion, and post-meiotic segregation (for details see Fig. 5).

We greatly appreciate the contributions of forerunners whose extensive works on the recombination of HIV-1 and other retroviruses provided the data upon which this study is based.

## Additional file

Additional file 1: Table S1. Breakpoint frequency of all 4 groups. **Table S2.** The screened hot spot and cold spot regions. **Table S3.** Corresponding breakpoint frequency and intersubtype genetic homology of each window. **dataset 1.** Base-pairing probability value of each site in hot spot regions. **dataset 2.** Base-pairing probability value of each site in cold spot regions. **Figure S1.** The minus-strand exchange model. **Figure S2.** Reverse transcription under limitation of RNase H activity. **Figure S3.** Recombination products from intrastrand BSs. **Figure S4.** Clustered BSs result in a high level of mutations near the crossover site. (DOCX 252 kb)
