# Supplementary material for: Analysis of HIV-1 intersubtype recombination breakpoints suggests region with high pairing probability may be a more fundamental factor than sequence similarity affecting HIV-1 recombination
Source: Virol J. 2016 Sep 21;13:156. doi: 10.1186/s12985-016-0616-1 (PMC5031261; doi:10.1186/s12985-016-0616-1)
Supplement: Additional file 1: Table S1. — Breakpoint frequency of all 4 groups. Table S2. The screened hot spot and cold spot regions. Table S3. Corresponding breakpoint frequency and intersubtype genetic homology of each window. dataset 1. Base-pairing probability value of each site in hot spot regions. dataset 2. Base-pairing probability value of each site in cold spot regions. Figure S1. The minus-strand exchange model. Figure S2. Reverse transcription under limitation of RNase H activity. Figure S3. Recombination products from intrastrand BSs. Figure S4. Clustered BSs result in a high level of mutations near the crossover site. (DOCX 252 kb) [file 12985_2016_616_MOESM1_ESM.docx]

**Supplementary Table 1** Breakpoint frequency of all 4 groups.

| Windows | Start*^a^* | End*^a^* | URFs from Africa | URFs from Asia | URFs from Europe | CRFs from around  the world |
| --- | --- | --- | --- | --- | --- | --- |
| 1 | 790 | 889 | 0 | 0 | 0 | 0 |
| 2 | 890 | 989 | 1 | 2 | 2 | 2 |
| 3 | 990 | 1089 | 4 | 5 | 0 | 2 |
| 4 | 1090 | 1189 | 23 | 11 | 11 | 2 |
| 5 | 1190 | 1289 | 7 | 10 | 8 | 5 |
| 6 | 1290 | 1389 | 2 | 1 | 0 | 1 |
| 7 | 1390 | 1489 | 2 | 0 | 5 | 2 |
| 8 | 1490 | 1589 | 3 | 0 | 2 | 0 |
| 9 | 1590 | 1689 | 6 | 0 | 1 | 0 |
| 10 | 1690 | 1789 | 7 | 2 | 4 | 2 |
| 11 | 1790 | 1889 | 10 | 18 | 7 | 2 |
| 12 | 1890 | 1989 | 5 | 6 | 3 | 2 |
| 13 | 1990 | 2089 | 5 | 9 | 5 | 4 |
| 14 | 2090 | 2189 | 15 | 12 | 2 | 11 |
| 15 | 2190 | 2289 | 25 | 5 | 7 | 5 |
| 16 | 2290 | 2389 | 5 | 11 | 0 | 3 |
| 17 | 2390 | 2489 | 7 | 2 | 9 | 5 |
| 18 | 2490 | 2589 | 10 | 13 | 9 | 8 |
| 19 | 2590 | 2689 | 5 | 7 | 2 | 5 |
| 20 | 2690 | 2789 | 7 | 10 | 1 | 3 |
| 21 | 2790 | 2889 | 9 | 15 | 4 | 10 |
| 22 | 2890 | 2989 | 10 | 7 | 4 | 5 |
| 23 | 2990 | 3089 | 8 | 6 | 3 | 3 |
| 24 | 3090 | 3189 | 6 | 7 | 5 | 9 |
| 25 | 3190 | 3289 | 20 | 16 | 6 | 10 |
| 26 | 3290 | 3389 | 8 | 8 | 1 | 4 |
| 27 | 3390 | 3489 | 3 | 3 | 6 | 2 |
| 28 | 3490 | 3589 | 4 | 7 | 1 | 4 |
| 29 | 3590 | 3689 | 1 | 3 | 4 | 3 |
| 30 | 3690 | 3789 | 12 | 10 | 5 | 7 |
| 31 | 3790 | 3889 | 4 | 4 | 6 | 1 |
| 32 | 3890 | 3989 | 3 | 0 | 1 | 2 |
| 33 | 3990 | 4089 | 6 | 6 | 5 | 1 |
| 34 | 4090 | 4189 | 20 | 4 | 6 | 10 |
| 35 | 4190 | 4289 | 15 | 14 | 6 | 5 |
| 36 | 4290 | 4389 | 10 | 6 | 5 | 0 |
| 37 | 4390 | 4489 | 5 | 5 | 4 | 1 |
| 38 | 4490 | 4589 | 5 | 4 | 3 | 1 |
| 39 | 4590 | 4689 | 12 | 5 | 1 | 3 |
| 40 | 4690 | 4789 | 7 | 8 | 2 | 0 |
| 41 | 4790 | 4889 | 6 | 5 | 2 | 1 |
| 42 | 4890 | 4989 | 16 | 5 | 8 | 6 |
| 43 | 4990 | 5089 | 13 | 7 | 4 | 3 |
| 44 | 5090 | 5189 | 7 | 6 | 2 | 4 |
| 45 | 5190 | 5289 | 4 | 2 | 1 | 0 |
| 46 | 5290 | 5389 | 13 | 0 | 4 | 3 |
| 47 | 5390 | 5489 | 7 | 3 | 0 | 4 |
| 48 | 5490 | 5589 | 16 | 11 | 9 | 3 |
| 49 | 5590 | 5689 | 13 | 4 | 0 | 5 |
| 50 | 5690 | 5789 | 14 | 8 | 5 | 5 |
| 51 | 5790 | 5889 | 9 | 17 | 6 | 4 |
| 52 | 5890 | 5989 | 23 | 13 | 14 | 7 |
| 53 | 5990 | 6089 | 41 | 15 | 14 | 8 |
| 54 | 6090 | 6189 | 16 | 4 | 2 | 5 |
| 55 | 6190 | 6289 | 35 | 13 | 19 | 3 |
| 56 | 6290 | 6389 | 37 | 19 | 9 | 11 |
| 57 | 6390 | 6489 | 10 | 10 | 8 | 7 |
| 58 | 6490 | 6589 | 8 | 6 | 9 | 2 |
| 59 | 6590 | 6689 | 6 | 0 | 3 | 1 |
| 60 | 6690 | 6789 | 1 | 2 | 1 | 1 |
| 61 | 6790 | 6889 | 4 | 0 | 5 | 3 |
| 62 | 6890 | 6989 | 7 | 1 | 1 | 3 |
| 63 | 6990 | 7089 | 6 | 0 | 5 | 3 |
| 64 | 7090 | 7189 | 4 | 1 | 3 | 2 |
| 65 | 7190 | 7289 | 5 | 0 | 6 | 2 |
| 66 | 7290 | 7389 | 3 | 3 | 1 | 1 |
| 67 | 7390 | 7489 | 5 | 1 | 1 | 0 |
| 68 | 7490 | 7589 | 7 | 3 | 3 | 4 |
| 69 | 7590 | 7689 | 3 | 7 | 4 | 2 |
| 70 | 7690 | 7789 | 3 | 3 | 5 | 2 |
| 71 | 7790 | 7889 | 1 | 2 | 1 | 1 |
| 72 | 7890 | 7989 | 1 | 0 | 1 | 2 |
| 73 | 7990 | 8089 | 5 | 0 | 0 | 0 |
| 74 | 8090 | 8189 | 2 | 0 | 2 | 0 |
| 75 | 8190 | 8289 | 21 | 10 | 5 | 5 |
| 76 | 8290 | 8389 | 27 | 15 | 8 | 10 |
| 77 | 8390 | 8489 | 10 | 4 | 5 | 4 |
| 78 | 8490 | 8589 | 17 | 31 | 2 | 8 |
| 79 | 8590 | 8689 | 25 | 7 | 18 | 10 |
| 80 | 8690 | 8789 | 17 | 11 | 3 | 8 |
| 81 | 8790 | 8889 | 13 | 19 | 5 | 6 |
| 82 | 8890 | 8989 | 10 | 9 | 1 | 3 |
| 83 | 8990 | 9089 | 12 | 29 | 4 | 5 |
| 84 | 9090 | 9189 | 18 | 7 | 5 | 5 |
| 85 | 9190 | 9289 | 8 | 0 | 2 | 1 |
| 86 | 9290 | 9389 | 13 | 3 | 5 | 5 |

*^a^* the position is relative to HXB2 (accession number K03455).

**Supplementary Table 2** The screened hot spot and cold spot regions.

| Windows | Hot/cold | Total breakpoint | Start position*^a^* | End position*^a^* | Gene locations |
| --- | --- | --- | --- | --- | --- |
| 4 | + | **47** | 1090 | 1189 | p17+p24 |
| 15 | + | **42** | 2190 | 2289 | p6 |
| 25 | + | **52** | 3190 | 3289 | Middle of p51 RT |
| 52 | + | **57** | 5890 | 5989 | Tat |
| 53 | + | **78** | 5990 | 6089 | tat+vpu |
| 55 | + | **70** | 6190 | 6289 | vpu+gp120 |
| 56 | + | **76** | 6290 | 6389 | vpu+gp120 |
| 76 | + | **60** | 8290 | 8389 | tat+rev+gp41 |
| 78 | + | **58** | 8490 | 8589 | rev |
| 79 | + | **60** | 8590 | 8689 | rev+gp41 |
| 81 | + | **43** | 8790 | 8889 | gp41+nef |
| 83 | + | **50** | 8990 | 9089 | nef |
| 1 | - | **0** | 790 | 889 | p17 |
| 2 | - | **7** | 890 | 989 | p17 |
| 6 | - | **4** | 1290 | 1389 | p24 |
| 8 | - | **5** | 1490 | 1589 | p24 |
| 9 | - | **7** | 1590 | 1689 | p24 |
| 32 | - | **6** | 3890 | 3989 | p15 RNase |
| 45 | - | **7** | 5190 | 5289 | vif |
| 60 | - | **5** | 6690 | 6789 | gp120 |
| 66 | - | **8** | 7290 | 7389 | gp120 |
| 67 | - | **7** | 7390 | 7489 | gp120 |
| 71 | - | **5** | 7790 | 7889 | gp41 |
| 72 | - | **4** | 7890 | 7989 | gp41 |
| 73 | - | **5** | 7990 | 8089 | gp41 |
| 74 | - | **4** | 8090 | 8189 | gp41 |
| 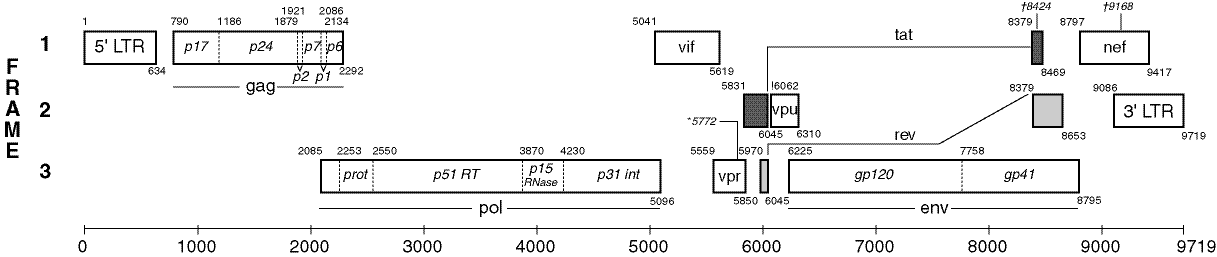 | | | | | |

*^a^* The positions were designated relative to HXB2 (Genbank accession no. K03455).

+ indicates hot spot regions.

- indicates cold spot regions.

The HIV-1 Gene Map is retrieved from the Los Alamos HIV sequence database (http://www.hiv.lanl.gov/content/sequence/HIV/MAP/landmark.html).

**Supplementary Table 3** Corresponding breakpoint frequency and intersubtype genetic homology of each window.

| Windows | Breakpoint frequency | Mean sequence identity |
| --- | --- | --- |
| 1 | 0 | 0.911454545 |
| 2 | 7 | 0.872363636 |
| 3 | 11 | 0.860909091 |
| 4 | 47 | 0.741381818 |
| 5 | 30 | 0.889818182 |
| 6 | 4 | 0.941454545 |
| 7 | 9 | 0.914363636 |
| 8 | 5 | 0.93 |
| 9 | 7 | 0.939090909 |
| 10 | 15 | 0.884727273 |
| 11 | 37 | 0.920545455 |
| 12 | 16 | 0.802272727 |
| 13 | 23 | 0.925090909 |
| 14 | 40 | 0.891636364 |
| 15 | 42 | 0.791818182 |
| 16 | 19 | 0.922363636 |
| 17 | 23 | 0.912181818 |
| 18 | 40 | 0.944545455 |
| 19 | 19 | 0.944363636 |
| 20 | 21 | 0.926181818 |
| 21 | 38 | 0.929636364 |
| 22 | 26 | 0.901454545 |
| 23 | 20 | 0.906727273 |
| 24 | 27 | 0.894545455 |
| 25 | 52 | 0.922909091 |
| 26 | 21 | 0.906909091 |
| 27 | 14 | 0.875090909 |
| 28 | 16 | 0.918727273 |
| 29 | 11 | 0.879090909 |
| 30 | 34 | 0.915090909 |
| 31 | 15 | 0.915818182 |
| 32 | 6 | 0.874 |
| 33 | 18 | 0.906909091 |
| 34 | 40 | 0.928363636 |
| 35 | 40 | 0.905836364 |
| 36 | 21 | 0.958 |
| 37 | 15 | 0.948545455 |
| 38 | 13 | 0.908727273 |
| 39 | 21 | 0.918 |
| 40 | 17 | 0.947636364 |
| 41 | 14 | 0.967272727 |
| 42 | 35 | 0.949272727 |
| 43 | 27 | 0.955636364 |
| 44 | 19 | 0.865818182 |
| 45 | 7 | 0.884309091 |
| 46 | 20 | 0.887454545 |
| 47 | 14 | 0.904363636 |
| 48 | 39 | 0.876727273 |
| 49 | 22 | 0.872181818 |
| 50 | 32 | 0.8964 |
| 51 | 36 | 0.875272727 |
| 52 | 57 | 0.860727273 |
| 53 | 78 | 0.7074 |
| 54 | 27 | 0.797454545 |
| 55 | 70 | 0.840381818 |
| 56 | 76 | 0.847272727 |
| 57 | 35 | 0.880909091 |
| 58 | 25 | 0.913454545 |
| 59 | 10 | 0.548927273 |
| 60 | 5 | 0.736854545 |
| 61 | 12 | 0.860363636 |
| 62 | 12 | 0.875454545 |
| 63 | 14 | 0.865818182 |
| 64 | 10 | 0.816127273 |
| 65 | 13 | 0.742818182 |
| 66 | 8 | 0.831818182 |
| 67 | 7 | 0.553618182 |
| 68 | 17 | 0.833090909 |
| 69 | 16 | 0.815509091 |
| 70 | 13 | 0.891454545 |
| 71 | 5 | 0.950909091 |
| 72 | 4 | 0.919272727 |
| 73 | 5 | 0.907272727 |
| 74 | 4 | 0.827090909 |
| 75 | 41 | 0.903272727 |
| 76 | 60 | 0.909454545 |
| 77 | 23 | 0.867090909 |
| 78 | 58 | 0.762436364 |
| 79 | 60 | 0.842 |
| 80 | 39 | 0.848909091 |
| 81 | 43 | 0.788218182 |
| 82 | 23 | 0.782109091 |
| 83 | 50 | 0.897636364 |
| 84 | 35 | 0.911454545 |
| 85 | 11 | 0.854 |
| 86 | 26 | 0.806181818 |

**Supplementary dataset 1** Base-pairing probability value of each site in hot spot regions.

| **Windows**  **Sites** | **4** | **15** | **25** | **52** | **53** | **55** | **56** | **76** | **78** | **79** | **81** | **83** |
| --- | --- | --- | --- | --- | --- | --- | --- | --- | --- | --- | --- | --- |
| Site 1 | 0.69 | 0 | 0.02 | 0.99 | 0.86 | 0.55 | 0.47 | 0.22 | 0.27 | 0.06 | 0.9 | 0.1 |
| Site 2 | 0.82 | 0 | 0.01 | 0.99 | 0.06 | 0.63 | 0.06 | 0.21 | 0.44 | 0.9 | 0.91 | 0.1 |
| Site 3 | 0.57 | 0 | 0.01 | 0.01 | 0.07 | 0.71 | 0.01 | 0.13 | 0.48 | 0.91 | 0.62 | 0.08 |
| Site 4 | 0.22 | 0 | 0.16 | 0.01 | 0.06 | 0.79 | 0.1 | 0.01 | 0.76 | 0.65 | 0.35 | 0.62 |
| Site 5 | 0.19 | 0 | 0.16 | 0.38 | 0.01 | 0.11 | 0.78 | 0.01 | 0.85 | 0.6 | 0.67 | 0.62 |
| Site 6 | 0.15 | 0 | 0.02 | 0.76 | 0.01 | 0.04 | 0.93 | 0 | 0.95 | 0.65 | 0.78 | 0.62 |
| Site 7 | 0.03 | 0 | 0.07 | 0.52 | 0.01 | 0.01 | 0.91 | 0 | 0.89 | 0.3 | 0.78 | 0.53 |
| Site 8 | 0.04 | 0 | 0.07 | 0.52 | 0 | 0.08 | 0.39 | 0.02 | 0.84 | 0.23 | 0.87 | 0.59 |
| Site 9 | 0.03 | 0 | 0.87 | 0.45 | 0 | 0.24 | 0.21 | 0.87 | 0.12 | 0 | 0.55 | 0.69 |
| Site 10 | 0.51 | 0 | 0.9 | 0 | 0 | 0.21 | 0.04 | 0.97 | 0.21 | 0 | 0.54 | 0.88 |
| Site 11 | 0.64 | 0 | 0.86 | 0.1 | 0.01 | 0.12 | 0.02 | 0.94 | 0.15 | 0.47 | 0.05 | 0.8 |
| Site 12 | 0.18 | 0 | 0.15 | 0.61 | 0.01 | 0.07 | 0.07 | 0.18 | 0.22 | 0.83 | 0.01 | 0.89 |
| Site 13 | 0.01 | 0 | 0.15 | 0.99 | 0 | 0.01 | 0.07 | 0.3 | 0.44 | 0.61 | 0.02 | 0.92 |
| Site 14 | 0.01 | 0 | 0 | 0.98 | 0 | 0.02 | 0.18 | 0.23 | 0.44 | 0.19 | 0.35 | 0.95 |
| Site 15 | 0.05 | 0 | 0 | 0.98 | 0.01 | 0.03 | 0.87 | 0.01 | 0.22 | 0.02 | 0.56 | 0.96 |
| Site 16 | 0.05 | 0 | 0 | 0.99 | 0.01 | 0.05 | 0.86 | 0.02 | 0.28 | 0 | 0.78 | 0.95 |
| Site 17 | 0.02 | 0 | 0 | 0.51 | 0 | 0.05 | 0.86 | 0.73 | 0.42 | 0 | 0.25 | 0.91 |
| Site 18 | 0.05 | 0.09 | 0 | 0.35 | 0 | 0.03 | 0.86 | 0.95 | 0.93 | 0 | 0.02 | 0.41 |
| Site 19 | 0.43 | 0.1 | 0 | 0.3 | 0 | 0.02 | 0.87 | 0.95 | 0.93 | 0 | 0.43 | 0.38 |
| Site 20 | 0.39 | 0.05 | 0 | 0.79 | 0 | 0.11 | 0.82 | 0.92 | 0.94 | 0 | 0.5 | 0.19 |
| Site 21 | 0 | 0.07 | 0.48 | 0.89 | 0 | 0.73 | 0.73 | 0.33 | 0.17 | 0 | 0.46 | 0.82 |
| Site 22 | 0 | 0.16 | 0.68 | 0.75 | 0 | 0.63 | 0.09 | 0.9 | 0.32 | 0.01 | 0.73 | 0.83 |
| Site 23 | 0 | 0.18 | 0.95 | 0.28 | 0 | 0.01 | 0.07 | 0.98 | 0.31 | 0.45 | 0.76 | 0.48 |
| Site 24 | 0.02 | 0.06 | 0.7 | 0.27 | 0 | 0.01 | 0.05 | 0.98 | 0.34 | 0.82 | 0.37 | 0.46 |
| Site 25 | 0.02 | 0.01 | 0.69 | 0.28 | 0 | 0.01 | 0.05 | 0.97 | 0.12 | 0.48 | 0.34 | 0.28 |
| Site 26 | 0 | 0.14 | 0.57 | 0.77 | 0 | 0.01 | 0.02 | 0.56 | 0.09 | 0.17 | 0.11 | 0.31 |
| Site 27 | 0 | 0.82 | 0.27 | 0.77 | 0 | 0.02 | 0 | 0.45 | 0.06 | 0.17 | 0.17 | 0.67 |
| Site 28 | 0 | 0.91 | 0.07 | 0.31 | 0 | 0.04 | 0 | 0.9 | 0.07 | 0.03 | 0.26 | 0.73 |
| Site 29 | 0 | 0.93 | 0.12 | 0.89 | 0 | 0.04 | 0 | 1 | 0.06 | 0 | 0.65 | 0.46 |
| Site 30 | 0 | 0.89 | 0.28 | 0.84 | 0 | 0.33 | 0 | 0.99 | 0.02 | 0 | 0.63 | 0.17 |
| Site 31 | 0 | 0.88 | 0.17 | 0.16 | 0 | 0.82 | 0 | 0.99 | 0.64 | 0 | 0.13 | 0.91 |
| Site 32 | 0 | 0.87 | 0.15 | 0.27 | 0 | 0.49 | 0 | 0.98 | 0.67 | 0 | 0.1 | 0.93 |
| Site 33 | 0.01 | 1 | 0.15 | 0.28 | 0 | 0.09 | 0 | 0.91 | 0.93 | 0 | 0.06 | 0.91 |
| Site 34 | 0.01 | 0.86 | 0.03 | 0.34 | 0.4 | 0.17 | 0.09 | 0.06 | 0.6 | 0 | 0.06 | 0.84 |
| Site 35 | 0 | 0.17 | 0.98 | 0.42 | 0.98 | 0.46 | 1 | 0.87 | 0.57 | 0 | 0.09 | 0.03 |
| Site 36 | 0 | 0.01 | 0.99 | 0.16 | 0.93 | 0.72 | 1 | 0.9 | 0.13 | 0 | 0.19 | 0.03 |
| Site 37 | 0 | 0.05 | 0.98 | 0.08 | 0.02 | 0.82 | 0.99 | 0.91 | 0.35 | 0 | 0.16 | 0.06 |
| Site 38 | 0 | 0.09 | 0.93 | 0.34 | 0.04 | 0.35 | 0.99 | 0.76 | 0.32 | 0.06 | 0.44 | 0.07 |
| Site 39 | 0 | 0.08 | 0.96 | 0.65 | 0.02 | 0.13 | 0.98 | 0.28 | 0.82 | 0.76 | 0.55 | 0.07 |
| Site 40 | 0.01 | 0.1 | 0.97 | 0.71 | 0.36 | 0.04 | 0.98 | 0.19 | 0.85 | 0.72 | 0.74 | 0.04 |
| Site 41 | 0.01 | 0.09 | 0.96 | 0.65 | 0.95 | 0.02 | 0.95 | 0.15 | 0.73 | 0.64 | 0.91 | 0.81 |
| Site 42 | 0 | 0.06 | 0.96 | 0.51 | 0.91 | 0.27 | 0.85 | 0.3 | 0.28 | 0.81 | 0.94 | 0.9 |
| Site 43 | 0 | 0.14 | 0.96 | 0.33 | 0.02 | 0.82 | 0.09 | 0.85 | 0.3 | 0.75 | 0.91 | 0.95 |
| Site 44 | 0 | 0.15 | 0.96 | 0.43 | 0.26 | 0.56 | 0.06 | 0.86 | 0.64 | 0.24 | 0.94 | 0.92 |
| Site 45 | 0 | 0.13 | 0.92 | 0.43 | 0.27 | 0.06 | 0.08 | 0.7 | 1 | 0.04 | 0.88 | 0.31 |
| Site 46 | 0 | 0.11 | 0.89 | 0.34 | 0.03 | 0.07 | 0.07 | 0.56 | 0.97 | 0.03 | 0.93 | 0.34 |
| Site 47 | 0 | 0.02 | 0.98 | 0.38 | 0.3 | 0.03 | 0.17 | 0.74 | 0.92 | 0 | 0.89 | 0.28 |
| Site 48 | 0 | 0.97 | 0.98 | 0.41 | 0.3 | 0.1 | 0.21 | 0.63 | 0.89 | 0.5 | 1 | 0.56 |
| Site 49 | 0 | 0.96 | 0.98 | 0.51 | 0.04 | 0.12 | 0.78 | 0.56 | 0.2 | 0.56 | 1 | 0.48 |
| Site 50 | 0 | 0 | 0.97 | 0.47 | 0.08 | 0.48 | 0.87 | 0.07 | 0.38 | 0.5 | 0 | 0.43 |
| Site 51 | 0 | 0 | 0.84 | 0.25 | 0.09 | 0.49 | 0.9 | 0.09 | 0.41 | 0.17 | 0 | 0.4 |
| Site 52 | 0 | 0 | 0.64 | 0.27 | 0.03 | 0.2 | 0.85 | 0.36 | 0.24 | 0.16 | 0 | 0.49 |
| Site 53 | 0 | 0 | 0.38 | 0.28 | 0.06 | 0.28 | 0.96 | 0.51 | 0.35 | 0.11 | 0.01 | 0.33 |
| Site 54 | 0 | 0.01 | 0.27 | 0.3 | 0.05 | 0.4 | 0.96 | 0.54 | 0.61 | 0.46 | 1 | 0.86 |
| Site 55 | 0 | 0.97 | 0.02 | 0.25 | 0.52 | 0.38 | 0.95 | 0.55 | 0.62 | 0.47 | 0.99 | 0.9 |
| Site 56 | 0.02 | 0.97 | 0.03 | 0.04 | 0.53 | 0.88 | 0.68 | 0.31 | 0.63 | 0.23 | 0.02 | 0.45 |
| Site 57 | 0.05 | 0.64 | 0.03 | 0.04 | 0.15 | 0.72 | 0.07 | 0.35 | 0.97 | 0.31 | 0 | 0.33 |
| Site 58 | 0.04 | 0.79 | 0.01 | 0 | 0.41 | 0.02 | 0.01 | 0.34 | 0.94 | 0.29 | 0 | 0.12 |
| Site 59 | 0.01 | 0.85 | 0.26 | 0.01 | 0.32 | 0.01 | 0.28 | 0.27 | 0.65 | 0.2 | 0 | 0.09 |
| Site 60 | 0 | 0.67 | 0.35 | 0 | 0.21 | 0.01 | 0.56 | 0.35 | 0.97 | 0.14 | 0.02 | 0.62 |
| Site 61 | 0 | 0.81 | 0.63 | 0 | 0.22 | 0.01 | 0.51 | 0.39 | 0.95 | 0.07 | 0.03 | 0.66 |
| Site 62 | 0 | 0.48 | 0.83 | 0.05 | 0.2 | 0.88 | 0.63 | 0.28 | 0.43 | 0.06 | 0.01 | 0.73 |
| Site 63 | 0.02 | 0.41 | 0.98 | 0.05 | 0.54 | 0.97 | 0.67 | 0.2 | 0.54 | 0.26 | 0.06 | 0.66 |
| Site 64 | 0.03 | 0.68 | 0.99 | 0.01 | 0.56 | 0.93 | 0.61 | 0.37 | 0.57 | 0.23 | 0.65 | 0.46 |
| Site 65 | 0.03 | 0.64 | 1 | 0.9 | 0.46 | 0.28 | 0.23 | 0.43 | 0.89 | 0.05 | 0.61 | 0.34 |
| Site 66 | 0.03 | 0.83 | 0.98 | 0.92 | 0.04 | 0.27 | 0.35 | 0.19 | 0.97 | 0.01 | 0.33 | 0.52 |
| Site 67 | 0 | 0.28 | 0.91 | 0.94 | 0.04 | 0.02 | 0.33 | 0.18 | 0.99 | 0.12 | 0.31 | 0.4 |
| Site 68 | 0 | 0.26 | 0.06 | 0.96 | 0.04 | 0.03 | 0.64 | 0.21 | 0.74 | 0.14 | 0 | 0.13 |
| Site 69 | 0 | 0.23 | 0.06 | 0.72 | 0.06 | 0.07 | 0.66 | 0.24 | 0.07 | 0.17 | 0 | 0.11 |
| Site 70 | 0 | 0.16 | 0.03 | 0.06 | 0.13 | 0.11 | 0.22 | 0.39 | 0.42 | 0.5 | 0 | 0.36 |
| Site 71 | 0 | 0.28 | 0.01 | 0.07 | 0.27 | 0.4 | 0.07 | 0.45 | 0.5 | 0.97 | 0 | 0.74 |
| Site 72 | 0 | 0.62 | 0.03 | 0.41 | 0.27 | 0.45 | 0.05 | 0.49 | 0.15 | 0.55 | 0 | 0.86 |
| Site 73 | 0 | 0.51 | 0.04 | 0.45 | 0.53 | 0.12 | 0.05 | 0.28 | 0.13 | 0 | 0 | 0.84 |
| Site 74 | 0 | 0.25 | 0.04 | 0.21 | 0.99 | 0.06 | 0.05 | 0.28 | 0.14 | 0.02 | 0 | 0.78 |
| Site 75 | 0 | 0.44 | 0.87 | 0.76 | 0.99 | 0.05 | 0.12 | 0.6 | 0.07 | 0.07 | 0 | 0.92 |
| Site 76 | 0.01 | 0.37 | 0.95 | 0.81 | 0 | 0.04 | 0.18 | 0.5 | 0.38 | 0.13 | 0 | 0.95 |
| Site 77 | 0.03 | 0.4 | 0.92 | 0.93 | 0 | 0.22 | 0.22 | 0.18 | 0.4 | 0.13 | 0 | 0.97 |
| Site 78 | 0.03 | 0.33 | 0.03 | 0.94 | 0 | 0.29 | 0.64 | 0.6 | 0.06 | 0.13 | 0 | 0.9 |
| Site 79 | 0.04 | 0.65 | 0.93 | 0.98 | 0 | 0.13 | 0.62 | 0.71 | 0.18 | 0.06 | 0.99 | 0.5 |
| Site 80 | 0.41 | 0.72 | 0.99 | 0.95 | 0 | 0.03 | 0.78 | 0.64 | 0.3 | 0.07 | 1 | 0 |
| Site 81 | 0.8 | 0.26 | 0.99 | 0.02 | 0 | 0.02 | 0.96 | 0.62 | 0.17 | 0.04 | 0.07 | 0 |
| Site 82 | 0.77 | 0.01 | 0.98 | 0.01 | 0 | 0.87 | 1 | 0.23 | 0.06 | 0.02 | 0.06 | 0.05 |
| Site 83 | 0.38 | 0.15 | 0.93 | 0.01 | 0 | 0.9 | 0.05 | 0.59 | 0.1 | 0.02 | 0.11 | 0.07 |
| Site 84 | 0.03 | 0.16 | 0.86 | 0.01 | 0 | 0.81 | 0.03 | 0.6 | 0.14 | 0.09 | 0.16 | 0.36 |
| Site 85 | 0.12 | 0.06 | 0.02 | 0.01 | 0 | 0.03 | 0.03 | 0.53 | 0.33 | 0.1 | 0.06 | 0.89 |
| Site 86 | 0.14 | 0.28 | 0.03 | 0.92 | 0 | 0.01 | 0.04 | 0.32 | 0.84 | 0.06 | 0.21 | 0.95 |
| Site 87 | 0.21 | 0.35 | 0.02 | 0.96 | 0 | 0 | 0.02 | 0.44 | 0.9 | 0.39 | 0.21 | 0.96 |
| Site 88 | 0.2 | 0.23 | 0.02 | 0.92 | 0 | 0 | 0.9 | 0.58 | 0.87 | 0.49 | 0.01 | 0.91 |
| Site 89 | 0.01 | 0.03 | 0.02 | 0.55 | 0 | 0 | 0.98 | 0.59 | 0.69 | 0.71 | 0 | 0.83 |
| Site 90 | 0.01 | 0.03 | 0.01 | 0.37 | 0 | 0 | 0.88 | 0.28 | 0.61 | 0.66 | 0.06 | 0.64 |
| Site 91 | 0.03 | 0.2 | 0.03 | 0.77 | 0 | 0.01 | 0.11 | 0.17 | 0.48 | 0.62 | 0.24 | 0.75 |
| Site 92 | 0.17 | 0.74 | 0.03 | 0.81 | 0 | 0.03 | 0.81 | 0.6 | 0.46 | 0.15 | 0.19 | 0.82 |
| Site 93 | 0.31 | 0.56 | 0.87 | 0.31 | 0 | 0.04 | 0.95 | 0.54 | 0.44 | 0.23 | 0.08 | 0.53 |
| Site 94 | 0.3 | 0.23 | 0.94 | 0.52 | 0 | 0.07 | 0.95 | 0.33 | 0.59 | 0.67 | 0.02 | 0.91 |
| Site 95 | 0.41 | 0.25 | 0.96 | 0.39 | 0.61 | 0.04 | 0.69 | 0.3 | 0.77 | 0.62 | 0.38 | 0.95 |
| Site 96 | 0.36 | 0.17 | 0.98 | 0.02 | 0.68 | 0.01 | 0.32 | 0.08 | 0.77 | 0.22 | 0.46 | 0.97 |
| Site 97 | 0.45 | 0.01 | 0.99 | 0.61 | 0.63 | 0.01 | 0.05 | 0.05 | 0.04 | 0.21 | 0.11 | 0.97 |
| Site 98 | 0.38 | 0.02 | 0.94 | 0.83 |  | 0.01 | 0.15 | 0.08 | 0.04 | 0.03 | 0.07 | 0.95 |
| Site 99 | 0.4 | 0.09 | 0.92 | 0.87 |  | 0.52 | 0.19 | 0.09 | 0.06 | 0.06 | 0.15 | 0.96 |
| Site 100 | 0.09 | 0.1 | 0.97 | 0.84 |  | 0.91 | 0.52 | 0.07 | 0.06 | 0.74 | 0.14 | 0.95 |

**Supplementary dataset 2** Base-pairing probability value of each site in cold spot regions.

| **Windows**  **Sites** | **1** | **2** | **6** | **8** | **9** | **32** | **45** | **60** | **66** | **67** | **71** | **72** | **73** | **74** |
| --- | --- | --- | --- | --- | --- | --- | --- | --- | --- | --- | --- | --- | --- | --- |
| Site 1 | 0.96 | 0.75 | 0.19 | 0.05 | 0.5 | 0.23 | 0.26 | 1 | 0.81 | 0 | 0.98 | 1 | 0.63 | 1 |
| Site 2 | 0.98 | 0.59 | 0.37 | 0.02 | 0.54 | 0.23 | 0.26 | 0.62 | 0.81 | 0 | 0.87 | 0.99 | 0.07 | 0.13 |
| Site 3 | 0.54 | 0.51 | 0.45 | 0.01 | 0.64 | 0.12 | 0.96 | 0.04 | 0.85 | 0.01 | 0.43 | 0.92 | 0.16 | 0 |
| Site 4 | 0.81 | 0.32 | 0.34 | 0.02 | 0.54 | 0.12 | 0.99 | 0.07 | 0.88 | 0.76 | 0.19 | 0.44 | 0.4 | 0 |
| Site 5 | 0.73 | 0.31 | 0.32 | 0.04 | 0.57 | 0.12 | 0.88 | 0.07 | 0.68 | 0.76 | 0.16 | 0.5 | 0.44 | 0 |
| Site 6 | 0.8 | 0.52 | 0.91 | 0.06 | 0.6 | 0 | 0.48 | 0 | 0.5 | 0.87 | 0.67 | 0.26 | 0.23 | 0.03 |
| Site 7 | 0.59 | 0.62 | 0.92 | 0.9 | 0.78 | 0 | 0.56 | 0 | 0.51 | 0.76 | 0.68 | 0.68 | 0.92 | 1 |
| Site 8 | 0.85 | 0.46 | 0.91 | 0.94 | 0.88 | 0 | 0.29 | 0.01 | 0.51 | 0.62 | 0.67 | 0.98 | 0.99 | 0.98 |
| Site 9 | 0.86 | 0.42 | 0.89 | 0.39 | 0.92 | 0.01 | 0.07 | 0.91 | 0 | 0.33 | 0.64 | 0.94 | 0.99 | 0.39 |
| Site 10 | 0.9 | 0.22 | 0.85 | 0.12 | 0.59 | 0.01 | 0 | 0.93 | 0.01 | 0 | 0.9 | 0.84 | 1 | 0.85 |
| Site 11 | 0.86 | 0.16 | 0.35 | 0.11 | 0.07 | 0 | 0.09 | 0.56 | 0.02 | 0.02 | 0.92 | 0.85 | 1 | 0.84 |
| Site 12 | 0.38 | 0.62 | 0.73 | 0.4 | 0.21 | 0 | 0.09 | 0.07 | 0.13 | 0.02 | 0.84 | 0.78 | 0.98 | 0.12 |
| Site 13 | 0.52 | 0.7 | 0.65 | 0.4 | 0.28 | 0.01 | 0.15 | 0.07 | 0.74 | 0.29 | 0.75 | 0.01 | 0.8 | 0 |
| Site 14 | 0.8 | 0.76 | 0.94 | 0.02 | 0.2 | 0.02 | 0.22 | 0.05 | 0.99 | 0.85 | 0.84 | 0.01 | 0.77 | 0 |
| Site 15 | 0.85 | 0.92 | 0.97 | 0.98 | 0.05 | 0.03 | 0.25 | 0.94 | 0.96 | 0.96 | 0.85 | 0.41 | 0.84 | 1 |
| Site 16 | 0.89 | 0.92 | 0.98 | 0.97 | 0.05 | 0.37 | 0.35 | 0.93 | 0.86 | 0.97 | 0.76 | 0.53 | 0.88 | 1 |
| Site 17 | 0.95 | 0.83 | 0.7 | 0.97 | 0.25 | 0.85 | 0.24 | 0.29 | 0.99 | 0.98 | 0.42 | 0.45 | 0.85 | 0.97 |
| Site 18 | 0.94 | 0.82 | 0.05 | 0.9 | 0.69 | 0.68 | 0.16 | 0 | 0.96 | 0 | 0.92 | 0.78 | 0.73 | 0.92 |
| Site 19 | 0.62 | 0.79 | 0.31 | 0.3 | 0.66 | 0.23 | 0.18 | 0 | 0.01 | 0 | 0.99 | 0.88 | 0.8 | 0.72 |
| Site 20 | 0.8 | 0.89 | 0.88 | 0.41 | 0.5 | 0.1 | 0.05 | 0 | 0.02 | 0 | 0.97 | 0.94 | 0.7 | 0.72 |
| Site 21 | 0.89 | 0.92 | 0.95 | 0.38 | 0.44 | 0.1 | 0.15 | 0 | 0.04 | 0 | 0.93 | 0.95 | 0.76 | 0.83 |
| Site 22 | 0.89 | 0.92 | 0.92 | 0.28 | 0.07 | 0.05 | 0.12 | 0.25 | 0.02 | 0 | 0.62 | 0.97 | 0.68 | 0.36 |
| Site 23 | 0.78 | 0.86 | 0.93 | 0.24 | 0.14 | 0.15 | 0.05 | 0.91 | 0 | 0 | 0.68 | 0.87 | 0.76 | 0.44 |
| Site 24 | 0.33 | 0.82 | 0.7 | 0.11 | 0.17 | 0.46 | 0.04 | 0.91 | 0.07 | 0 | 0.75 | 0.05 | 0.5 | 0.47 |
| Site 25 | 0.3 | 0.62 | 0.65 | 0.22 | 0.14 | 0.45 | 0.05 | 0 | 0.11 | 0 | 0.61 | 0.06 | 0.52 | 0.33 |
| Site 26 | 0.18 | 0.39 | 0.55 | 0.38 | 0.24 | 0.03 | 0.91 | 0 | 0.48 | 0 | 0.39 | 0.01 | 0.93 | 0.45 |
| Site 27 | 0.13 | 0.33 | 0.34 | 0.35 | 0.46 | 0.16 | 0.92 | 0 | 0.77 | 0 | 0.32 | 0.01 | 0.99 | 0.28 |
| Site 28 | 0.2 | 0.41 | 0.14 | 0.21 | 0.45 | 0.16 | 0.89 | 0.02 | 0.87 | 0 | 0.93 | 0 | 0.99 | 0.07 |
| Site 29 | 0.27 | 0.45 | 0.14 | 0.14 | 0.7 | 0.08 | 0.01 | 0.99 | 0.25 | 0 | 0.84 | 0.85 | 1 | 0.05 |
| Site 30 | 0.28 | 0.58 | 0.2 | 0.12 | 0.6 | 0.09 | 0.01 | 0.99 | 0 | 0 | 0.97 | 1 | 1 | 0.07 |
| Site 31 | 0.3 | 0.54 | 0.23 | 0.08 | 0.63 | 0.54 | 0.01 | 0 | 0.01 | 0 | 0.99 | 1 | 0.99 | 0.09 |
| Site 32 | 0.35 | 0.3 | 0.61 | 0.19 | 0.44 | 0.5 | 0 | 0 | 0.02 | 0 | 0.98 | 0.98 | 0.97 | 0.17 |
| Site 33 | 0.36 | 0.27 | 0.66 | 0.19 | 0.42 | 0.59 | 0.04 | 0 | 0.61 | 0 | 1 | 0.89 | 0.7 | 0.15 |
| Site 34 | 0.34 | 0.29 | 0.2 | 0.04 | 0.3 | 0.44 | 1 | 0 | 0.76 | 0 | 0.95 | 0.78 | 0.62 | 0.05 |
| Site 35 | 0.51 | 0.31 | 0.07 | 0.06 | 0.41 | 0.05 | 0.97 | 0 | 0.68 | 0 | 0.86 | 0.29 | 0.58 | 0.04 |
| Site 36 | 0.64 | 0.34 | 0.02 | 0.1 | 0.48 | 0.77 | 0.01 | 0.99 | 0.23 | 0 | 0.04 | 0.47 | 0.02 | 0.13 |
| Site 37 | 0.85 | 0.4 | 0.02 | 0.06 | 0.54 | 0.78 | 0.01 | 0.99 | 0.18 | 0 | 0.03 | 0.72 | 0.01 | 0.64 |
| Site 38 | 0.84 | 0.28 | 0.01 | 0.16 | 0.35 | 0.03 | 0 | 0.02 | 0.06 | 0 | 0 | 0.79 | 0.04 | 0.84 |
| Site 39 | 0.56 | 0.18 | 0.1 | 0.18 | 0.11 | 0.04 | 0 | 0 | 0 | 0 | 0 | 0.56 | 0.33 | 0.85 |
| Site 40 | 0.41 | 0.16 | 0.22 | 0.92 | 0.08 | 0.05 | 0 | 0 | 0 | 0 | 0 | 0.18 | 0.44 | 0.33 |
| Site 41 | 0.42 | 0.22 | 0.4 | 0.92 | 0.18 | 0.05 | 0.97 | 0 | 0.71 | 0 | 0.85 | 0.32 | 0.52 | 0.12 |
| Site 42 | 0.43 | 0.35 | 0.29 | 0.09 | 0.29 | 0.04 | 0.97 | 0 | 0.97 | 0 | 0.94 | 0.32 | 0.33 | 0.12 |
| Site 43 | 0.44 | 0.45 | 0.01 | 0.11 | 0.2 | 0.05 | 0.01 | 0 | 0.47 | 0 | 0.99 | 0.86 | 0.89 | 0.14 |
| Site 44 | 0.5 | 0.46 | 0.01 | 0.14 | 0.21 | 0.05 | 0 | 0 | 0.13 | 0 | 0.99 | 0.93 | 1 | 0.17 |
| Site 45 | 0.99 | 0.64 | 0.02 | 0.96 | 0.57 | 0.03 | 0.05 | 0 | 0.77 | 0 | 1 | 1 | 1 | 0.71 |
| Site 46 | 0.98 | 0.64 | 0.02 | 0.97 | 0.79 | 0.01 | 0.06 | 0 | 0.87 | 0 | 0.98 | 1 | 1 | 0.84 |
| Site 47 | 0.39 | 0.49 | 0.22 | 0.59 | 0.86 | 0.02 | 0.05 | 0 | 0.86 | 0 | 0.12 | 1 | 1 | 0.81 |
| Site 48 | 0.24 | 0.51 | 0.22 | 0.41 | 0.86 | 0.02 | 0.65 | 0 | 0.43 | 0 | 0.85 | 1 | 1 | 0.17 |
| Site 49 | 0.12 | 0.49 | 0.28 | 0.44 | 0.62 | 0.03 | 0.83 | 0 | 0.28 | 0 | 0.88 | 0.97 | 1 | 0.13 |
| Site 50 | 0.17 | 0.47 | 0.43 | 0.13 | 0.42 | 0.07 | 0.68 | 0 | 0.28 | 0 | 0.5 | 1 | 1 | 0.14 |
| Site 51 | 0.38 | 0.86 | 0.68 | 0.09 | 0.2 | 0.07 | 0.33 | 0 | 0.69 | 0 | 0.63 | 0.99 | 1 | 0.26 |
| Site 52 | 0.43 | 0.85 | 0.45 | 0.41 | 0.19 | 0.01 | 0.14 | 0 | 0.77 | 0 | 0.56 | 0.01 | 1 | 0.27 |
| Site 53 | 0.24 | 0.49 | 0.28 | 0.41 | 0.21 | 0.01 | 0.14 | 0.15 | 0.24 | 0 | 0.61 | 0 | 0.98 | 0.11 |
| Site 54 | 0.39 | 0.4 | 0.25 | 0.05 | 0.29 | 0.01 | 0.12 | 0.5 | 0.08 | 0 | 0.79 | 0 | 0.22 | 0.14 |
| Site 55 | 0.41 | 0.27 | 0.25 | 0.05 | 0.26 | 0.01 | 0.17 | 0.39 | 0.11 | 0 | 0.72 | 0 | 0.78 | 0.99 |
| Site 56 | 0.43 | 0.17 | 0.19 | 0.07 | 0.4 | 0.05 | 0.37 | 0.28 | 0.11 | 0 | 0.26 | 0 | 0.81 | 0.96 |
| Site 57 | 0.51 | 0.09 | 0.01 | 0.08 | 0.49 | 0.28 | 0.26 | 0.27 | 0.07 | 0 | 0.88 | 0 | 0.5 | 0.36 |
| Site 58 | 0.38 | 0.09 | 0.01 | 0.51 | 0.42 | 0.3 | 0.52 | 0.89 | 0.06 | 0 | 0.99 | 0 | 0.73 | 0.17 |
| Site 59 | 0.16 | 0.22 | 0.62 | 0.56 | 0.22 | 0.29 | 0.65 | 0.88 | 0.01 | 0 | 1 | 0.01 | 0.82 | 0.2 |
| Site 60 | 0.16 | 0.24 | 0.95 | 0.54 | 0.25 | 0.49 | 0.26 | 0 | 0.02 | 0 | 1 | 0.99 | 0.96 | 0.19 |
| Site 61 | 0.57 | 0.77 | 0.98 | 0.56 | 0.29 | 0.51 | 0.12 | 0.07 | 0.33 | 0 | 0.99 | 1 | 0.99 | 0.23 |
| Site 62 | 0.71 | 0.82 | 0.87 | 0.54 | 0.48 | 0.51 | 0.07 | 0.07 | 0.71 | 0 | 0.98 | 0.97 | 1 | 0.15 |
| Site 63 | 0.63 | 0.16 | 0.96 | 0.07 | 0.51 | 0.42 | 0.8 | 0 | 0.72 | 0 | 0.84 | 1 | 0.99 | 0.07 |
| Site 64 | 0.61 | 0.21 | 0.98 | 0.07 | 0.23 | 0.76 | 0.94 | 0.01 | 0.63 | 0 | 0 | 1 | 0.99 | 0.02 |
| Site 65 | 0.23 | 0.27 | 0.95 | 0.07 | 0.33 | 0.78 | 0.71 | 0.04 | 0.69 | 0 | 0 | 0.99 | 0.97 | 0.07 |
| Site 66 | 0.24 | 0.28 | 0.79 | 0.22 | 0.35 | 0.8 | 0.03 | 0.04 | 0.75 | 0 | 0 | 0.99 | 0.41 | 0.08 |
| Site 67 | 0.5 | 0.27 | 0.37 | 0.66 | 0.34 | 0.8 | 0.01 | 0.93 | 0.83 | 0 | 0 | 0.79 | 0.32 | 0.71 |
| Site 68 | 0.49 | 0.24 | 0.42 | 0.49 | 0.2 | 0.79 | 0.02 | 0.95 | 0.49 | 0 | 0.01 | 0.78 | 0.29 | 0.76 |
| Site 69 | 0.28 | 0.88 | 0.22 | 0.75 | 0.17 | 0.27 | 0.02 | 0.03 | 0.96 | 0 | 0.85 | 0.21 | 0.28 | 0.76 |
| Site 70 | 0.26 | 0.89 | 0.18 | 0.52 | 0.12 | 0.95 | 0.69 | 0.01 | 0.96 | 0 | 0.98 | 0.43 | 0.59 | 0.17 |
| Site 71 | 0.22 | 0.25 | 0.11 | 0.37 | 0.12 | 0.96 | 0.93 | 0.37 | 0.88 | 0.8 | 0.99 | 0.5 | 0.71 | 0.18 |
| Site 72 | 0.2 | 0.56 | 0.19 | 0.38 | 0.1 | 0.82 | 0.9 | 0.96 | 0.92 | 0.82 | 1 | 0.61 | 0.74 | 0.22 |
| Site 73 | 0.35 | 0.52 | 0.16 | 0.45 | 0.11 | 0.52 | 0.35 | 0.63 | 0.82 | 0.06 | 1 | 0.54 | 0.3 | 0.08 |
| Site 74 | 0.34 | 0.15 | 0.41 | 0.5 | 0.21 | 0.49 | 0.32 | 0.28 | 0.82 | 0.02 | 0.99 | 0.86 | 0.1 | 0.09 |
| Site 75 | 0.21 | 0.36 | 0.64 | 0.76 | 0.26 | 0.51 | 0.23 | 0.85 | 0.79 | 0.78 | 0.84 | 0.96 | 0.81 | 0.07 |
| Site 76 | 0.28 | 0.34 | 0.76 | 0.77 | 0.26 | 0.52 | 0.21 | 0.84 | 0.07 | 0.83 | 0.76 | 0.96 | 0.86 | 0.59 |
| Site 77 | 0.35 | 0.08 | 0.52 | 0.62 | 0.63 | 0.47 | 0.17 | 0.13 | 0.01 | 0.07 | 0.87 | 0.8 | 0.84 | 0.68 |
| Site 78 | 0.29 | 0.18 | 0.27 | 0.07 | 0.58 | 0.49 | 0.08 | 0.16 | 0.01 | 0.12 | 0.93 | 0.3 | 0.43 | 0.94 |
| Site 79 | 0.27 | 0.25 | 0.49 | 0.1 | 0.27 | 0.51 | 0.23 | 0.14 | 0.03 | 0.14 | 0.94 | 0.79 | 0.48 | 0.89 |
| Site 80 | 0.28 | 0.21 | 0.53 | 0.99 | 0.16 | 0.41 | 0.35 | 0 | 0.72 | 0.1 | 0.93 | 0.9 | 0.59 | 0.88 |
| Site 81 | 0.24 | 0.75 | 0.38 | 0.95 | 0.15 | 0.43 | 0.2 | 0 | 0.81 | 0.92 | 0.99 | 0.92 | 0.68 | 0.09 |
| Site 82 | 0.21 | 0.75 | 0.27 | 0.98 | 0.04 | 0.86 | 0.07 | 0.01 | 0.89 | 0.9 | 0.99 | 0.56 | 0.56 | 0.09 |
| Site 83 | 0.17 | 0.06 | 0.27 | 0.95 | 0.07 | 0.87 | 0.07 | 0.84 | 0.97 | 0.01 | 0.92 | 0.8 | 0.87 | 0.09 |
| Site 84 | 0.27 | 0 | 0.14 | 0.87 | 0.43 | 0.83 | 0.35 | 0.84 | 0.99 | 0.19 | 0.12 | 0.81 | 0.64 | 0.09 |
| Site 85 | 0.38 | 0.01 | 0.03 | 0.03 | 0.72 | 0.09 | 0.99 | 0.42 | 0.99 | 0.47 | 0.06 | 0.63 | 0.47 | 0.09 |
| Site 86 | 0.41 | 0.02 | 0.76 | 0.03 | 0.84 | 0.01 | 0.98 | 0.42 | 0.85 | 0.58 | 0.91 | 0.48 | 0.18 | 0.09 |
| Site 87 | 0.45 | 0.01 | 0.85 | 0.02 | 0.29 | 0.02 | 0.52 | 0.35 | 0.65 | 0.78 | 0.98 | 0.6 | 0.53 | 0.1 |
| Site 88 | 0.27 | 0.09 | 0.83 | 0 | 0.91 | 0.02 | 0.03 | 0 | 0.9 | 0.44 | 1 | 0.35 | 0.54 | 0.22 |
| Site 89 | 0.07 | 0.15 | 0.44 | 0.01 | 0.84 | 0.01 | 0.08 | 0 | 0.76 | 0.01 | 1 | 0.36 | 0.36 | 0.92 |
| Site 90 | 0.14 | 0.33 | 0.46 | 0.87 | 0.76 | 0.1 | 0.08 | 0 | 0.04 | 0.01 | 1 | 0.23 | 0.02 | 0.85 |
| Site 91 | 0.27 | 0.69 | 0.31 | 0.94 | 0.05 | 0.93 | 0.41 | 0 | 0.03 | 0 | 1 | 0.24 | 0 | 0.05 |
| Site 92 | 0.51 | 0.4 | 0.26 | 0.98 | 0.1 | 0.97 | 0.75 | 0 | 0.03 | 0.27 | 0.9 | 0.54 | 0 | 0.18 |
| Site 93 | 0.41 | 0.06 | 0.73 | 0.99 | 0.13 | 0.88 | 0.69 | 0 | 0.01 | 0.78 | 0 | 0.84 | 0.07 | 0.18 |
| Site 94 | 0.34 | 0.07 | 0.59 | 0.99 | 0.63 | 0.6 | 0.02 | 0 | 0.01 | 0.86 | 0 | 0.97 | 0.99 | 0.04 |
| Site 95 | 0.33 | 0.08 | 0.56 | 0.1 | 0.89 | 0.71 | 0 |  | 0.03 | 0.57 | 0 | 0.88 | 1 | 0.05 |
| Site 96 | 0.08 | 0.06 | 0.18 | 0.05 | 0.95 | 0.79 | 0.03 |  | 0.1 | 0.52 | 0 | 0.86 | 0.01 | 0.05 |
| Site 97 | 0.1 | 0.05 | 0.01 | 0.05 | 0.95 | 0.76 | 0.09 |  | 0.07 | 0.16 | 0.9 | 0.88 | 0 | 0.04 |
| Site 98 | 0.09 | 0.27 | 0.26 | 0.05 | 0.94 | 0.4 | 0.11 |  | 0.06 | 0.09 | 1 | 0.94 | 0 | 0.04 |
| Site 99 | 0.15 | 0.26 | 0.65 | 0.06 | 0.97 | 0.4 | 0.32 |  | 0.06 | 0.04 | 1 | 0.98 | 0 | 0.04 |
| Site 100 | 0.5 | 0.06 | 0.76 | 0.44 | 0.94 | 0.45 | 0.64 |  | 0 | 0.04 | 1 | 0.76 | 0.94 | 0.04 |


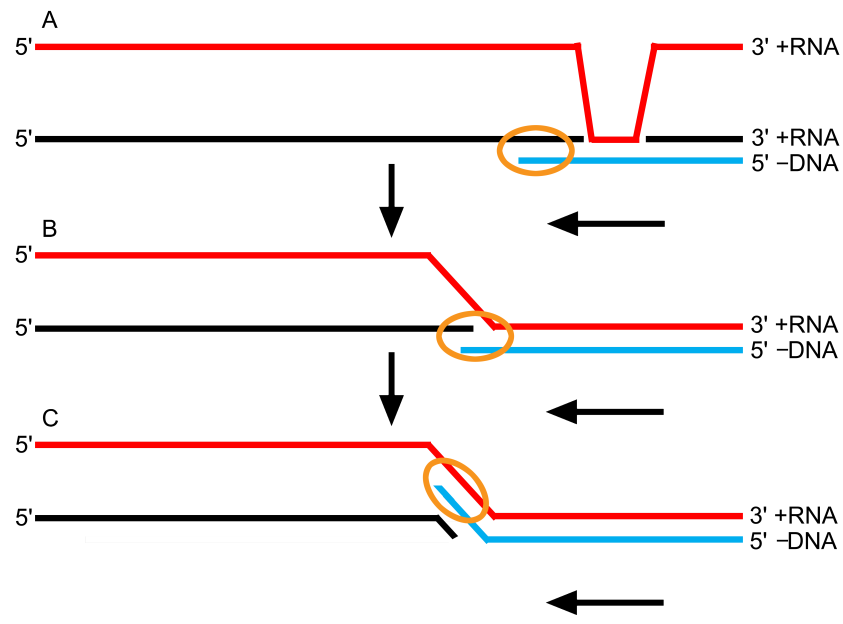


**Supplementary Figure 1. The minus-strand exchange model.**

(A) RNase H mediated template degradation exposes nascent minus-strand DNA, allowing base pairs between DNA synthesized on the donor template and complementary regions of the acceptor template.

(B) DNA strand realignment by branch migration follows, and the acceptor template “catches up” with the elongating RT.

(C) RT translocates and resumes DNA synthesis on the acceptor template. The black lines represent donor templates; the red lines represent acceptor templates; the blue line represents a nascent DNA (primer). HIV-1 RT is represented by the gold oval; arrows indicate the direction of DNA synthesis.


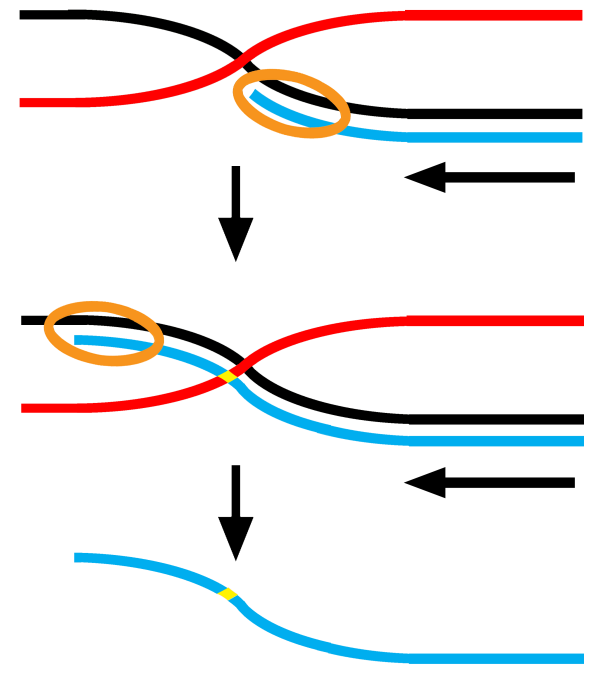


**Supplementary Figure 2.Reverse transcription under limitation of RNase H activity.**

When RNase H activity is inhibited, the hybrid duplex composed of the nascent DNA and donor strand is preserved well and the nascent DNA strand's base pairing with the acceptor template would be impossible, even if there is high donor-acceptor homology. Moreover, the stability of the BS increases due to inhibition of donor template degradation. Therefore, RT tends to advance in the original direction, with mutations becoming more likely.


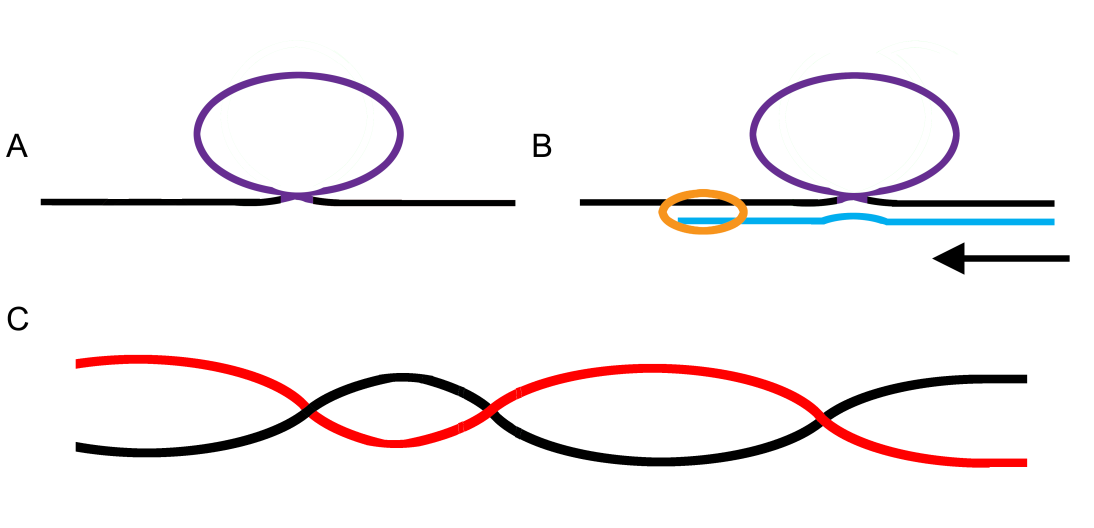


**Supplementary Figure 3.Recombination products from intrastrand BSs.**

(A) BS from intrastrand relative motion. The purple is used to distinguish gene regions.

(B) When RT encounters this category of BS, it transfers to the left part of the BS, and the purple gene is not replicated. The flanking genes are combined directly, resulting in deletion of the purple region.

(C) Interstrand intertwinement would reduce intrastrand BSs and therefore reduce the occurrence of the resulting deletions.


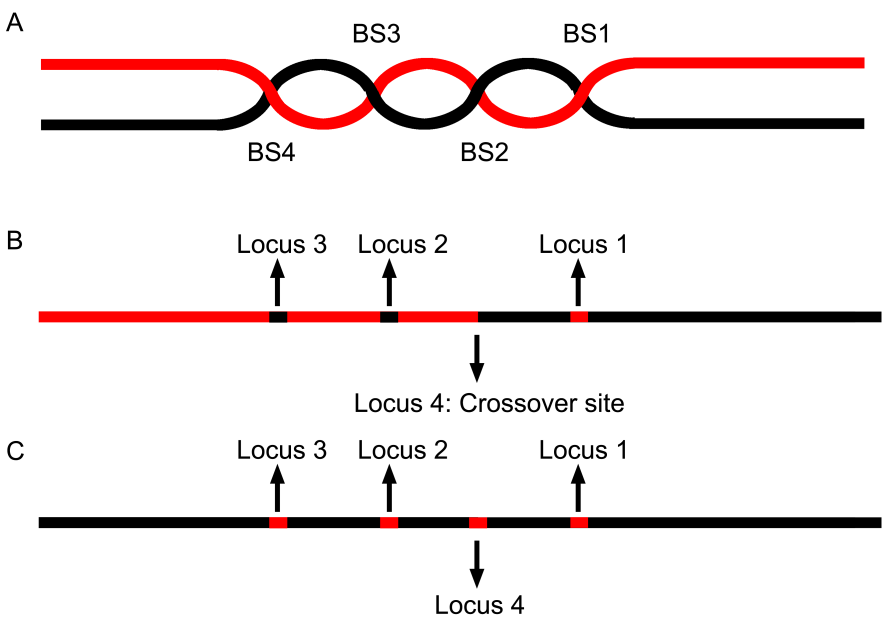


**Supplementary Figure 4.Clustered BSs result in a high level of mutations near the crossover site.**

(A) Here4 BSs form along a region due to consecutive intertwinement (BS1, BS2, BS3, and BS4).

(B) In this hypothetical scenario, reverse transcription lead to “mutations” atBS1, BS3, and BS4 in progeny at locus 1, locus 2, and locus 3, respectively.At theBS2 recombination is introduced in progeny at locus 4. This scenario would result in a high level of mutations near the crossover site.

(C) In a hypothetical scenario where BS1, BS2, BS3, and BS4 all cause “mutations” in progeny, only a series of mutations are observed.
